# Modeling the Role of the Microbiome in Evolution

**DOI:** 10.3389/fphys.2018.01836

**Published:** 2018-12-20

**Authors:** Saúl Huitzil, Santiago Sandoval-Motta, Alejandro Frank, Maximino Aldana

**Affiliations:** ^1^Instituto de Ciencias Físicas, Universidad Nacional Autónoma de México, Cuernavaca, Mexico; ^2^Centro de Ciencias de la Complejidad, Universidad Nacional Autónoma de México, Mexico City, Mexico; ^3^Instituto Nacional de Medicina Genómica, Mexico City, Mexico; ^4^Consejo Nacional de Ciencia y Tecnología, Cátedras CONACyT, Mexico City, Mexico; ^5^Instituto de Ciencias Nucleares, Universidad Nacional Autónoma de México, Mexico City, Mexico; ^6^Member of El Colegio Nacional, Mexico City, Mexico

**Keywords:** holobiont, coevolution, microbiome, symbiosis, complex networks, adaptability, microbiota diversity

## Abstract

There is undeniable evidence showing that bacteria have strongly influenced the evolution and biological functions of multicellular organisms. It has been hypothesized that many host-microbial interactions have emerged so as to increase the adaptive fitness of the *holobiont* (the host plus its microbiota). Although this association has been corroborated for many specific cases, general mechanisms explaining the role of the microbiota in the evolution of the host are yet to be understood. Here we present an evolutionary model in which a network representing the host adapts in order to perform a predefined function. During its adaptation, the host network (HN) can interact with other networks representing its microbiota. We show that this interaction greatly accelerates and improves the adaptability of the HN without decreasing the adaptation of the microbial networks. Furthermore, the adaptation of the HN to perform several functions is possible only when it interacts with many different bacterial networks in a specialized way (each bacterial network participating in the adaptation of one function). Disrupting these interactions often leads to non-adaptive states, reminiscent of dysbiosis, where none of the networks the holobiont consists of can perform their respective functions. By considering the holobiont as a unit of selection and focusing on the adaptation of the host to predefined but arbitrary functions, our model predicts the need for specialized diversity in the microbiota. This structural and dynamical complexity in the holobiont facilitates its adaptation, whereas a homogeneous (non-specialized) microbiota is inconsequential or even detrimental to the holobiont's evolution. To our knowledge, this is the first model in which symbiotic interactions, diversity, specialization and dysbiosis in an ecosystem emerge as a result of coevolution. It also helps us understand the emergence of complex organisms, as they adapt more easily to perform multiple tasks than non-complex ones.

## Introduction

It has been firmly established during the last decade that the microbiota of a multicellular host strongly influences its evolution and adaptation (Ley et al., 2008; Zilber-Rosenberg and Rosenberg, 2008; Rosenberg et al., 2010; Brucker and Bordenstein, 2013; Andrew et al., 2016; Rosenberg and Zilber-Rosenberg, 2016; Sharpton, 2018). In turn, the host's ability to interact with other organisms and modify its environment to its advantage can guide the composition of its microbiota (Mai, 2004; Spor et al., 2011; Yatsunenko et al., 2012; Moeller et al., 2014). For instance, the human microbiota plays an important role in many fundamental physiological functions, such as the development of the immune system (Hooper et al., 2012), degradation of fiber and metabolization of fats and carbohydrates (Krajmalnik-Brown et al., 2012), regulation of bone density (McCabe et al., 2015), metabolization of drugs (Wilson and Nicholson, 2017) and control of infections by pernicious bacteria like *Clostridium difficile* (Rupnik et al., 2009; Van Nood et al., 2013; Seekatz and Young, 2014). A state of imbalance in the human microbiota, known as dysbiosis, has been correlated with diseases (Cho and Blaser, 2012) such as obesity (Ley et al., 2006b; Ley, 2010), inflammatory bowel disease (Morgan et al., 2012; Halfvarson et al., 2017), cancer (Farrell et al., 2011; Zackular et al., 2013; Francescone et al., 2014; Sears and Garrett, 2014; Contreras et al., 2016; Yang et al., 2017) and even neurological disorders as schizophrenia and autism (Gonzalez et al., 2011; Rogers et al., 2016). The system consisting of the host and its microbiota, known as *holobiont*, exhibits the unequivocal existence of symbiotic relationships between microbes and multicellular organisms (Theis et al., 2016). Given these complex interdependencies, the holobiont has been proposed to function as a single evolutionary unit (Gilbert et al., 2012; Gordon et al., 2013; Guerrero et al., 2013; Bordenstein and Theis, 2015; Theis et al., 2016; Roughgarden et al., 2018). This is because environmental changes may impose selective pressures on the host which in turn will affect its microbiota (for a recent review see Roughgarden et al., 2018). In the light of these findings, it has been suggested that evolutionary theories have to be either reformulated or expanded in order to account for the adaptability of the holobiont as an evolutionary unit (Laland et al., 2014; Ereshefsky and Pedroso, 2015; Van Opstal and Bordenstein, 2015; Sandoval-Motta et al., 2017). Whether the holobiont is or is not an evolutionary unit is still a matter of debate (Moran and Sloan, 2015; Douglas and Werren, 2016; Doolittle and Booth, 2017; Doolittle and Inkpen, 2018). However, here we show that selective pressures applied to the host and its associated microbes taken as whole, can help us explain how symbiotic relationships in holobionts arose and are currently maintained.

Examples of the influence that microorganisms have had on the adaptation of their hosts range from cases in which microbes help the host to perform specific non-essential functions, to cases in which microbes have completely substituted essential functions of the host (Sagan, 1967; Zilber-Rosenberg and Rosenberg, 2008; Queller and Strassmann, 2016; Roughgarden et al., 2018). Nevertheless, the specific mechanisms by which this influence is carried on are not yet known. Particularly, what are the general benefits that the microbiota provides to the host during its evolution is still an open question. A possible answer to it is that the adaptation time of the host to face new environmental challenges is considerably reduced due to the great diversity and plasticity of its microbiota (Zilber-Rosenberg and Rosenberg, 2008; Rosenberg and Zilber-Rosenberg, 2016). This hypothesis assumes that the emergence of strong symbiotic relationships between the host and its microbiota occurs at the genetic and metabolic levels, for only in this way changes occurring in the microbiota can rapidly propagate to the host's metabolism and affect its adaptability. Indeed, recent evidence shows that the microbiota can regulate metabolic pathways and gene expression patterns of its host, and due to this interaction the host can properly perform cell differentiation, tissue formation, nutrition and other important functions (Hooper et al., 2001; Rawls et al., 2004; Bates et al., 2006; Shin et al., 2011; Nicholson et al., 2012; Camp et al., 2014).

It has been proposed that natural selection operating at the Host-level promotes stable and redundant microbial societies, whereas selection operating at the microbial level promotes functional specialization of their component species (Ley et al., 2006a). Despite all the knowledge we have now on human associated microbial communities, we still do not fully understand the evolutionary forces behind the diversity observed in our microbiota. On the one hand, the most abundant ecological relationship between microbial species is competition (Foster and Bell, 2012; Coyte et al., 2015; Moran and Sloan, 2015; Douglas and Werren, 2016), which often leads to uniform microbial communities where just a few species dominate the whole environment. On the other hand, it has been shown that purely mutualistic interactions lead to unstable communities as their diversity increases. These observations are at odds with the great diversity and stability observed in the microbiota of most plants and animals. Maintaining this diversity is fundamental for the survivability of the host, as it is known that a loss in the microbiota's diversity may produce severe dysbiosis that can result in host diseases or even death (Blaser and Falkow, 2009; Turnbaugh and Stintzi, 2011; Cho and Blaser, 2012; Fernández et al., 2013; Lloyd-Price et al., 2016; Blaser, 2017). An observation that circumvents this caveat is that multicellular organisms have developed different mechanisms to maintain the equilibrium between its diverse microbial communities. These mechanisms tend to compartmentalize the microbes in separate niches while reducing the interactions between microbes in the same niche (Grice and Segre, 2011; Donaldson et al., 2015; Deines et al., 2017; Tropini et al., 2017; Roughgarden et al., 2018). Understanding why microbial diversity is necessary for the evolution and adaptation of the host, and why disease arises when such diversity is lost, is a fundamental question with still no definitive answer.

To address these questions, we adopt the hypothesis that the holobiont constitutes a unit of selection in evolution and explore its consequences. We present an evolutionary population model in which the biological functions of organisms are encoded in the Boolean dynamics of regulatory networks. In our model, a host is represented as a Boolean network that needs to evolve in order to adequately perform a predefined task (or function). This is equivalent to the host acquiring a new phenotype in order to cope with a new environmental challenge. A population of such host networks is evolved in a way that each host network can establish regulatory interactions with a set of microbial species, each one represented also by a network. The main difference between the microbial and host networks is that due to the faster duplication rates of microbes, the generation of mutants is at least one order of magnitude larger in the microbial networks than in the host. Mutants, as explained in detail in the Materials and Methods section (M&M), are simulated by rewiring the connections of their network, or by altering their functionality. As we are dealing with evolutionary dynamics, it is important to mention that we will only consider host-microbe interactions that can be transmitted across generations. This is based on the fact that in many species, parents directly transmit their microbiota to their offspring or they construct environments with a stable microbial composition that bias the microbial composition of their progeny (Rosenberg et al., 2010; Fitzpatrick, 2014). Another important assumption in our model is the persistence across generations of the host-microbe interactions developed throughout the evolution of the holobiont, which is a necessary condition for natural selection to operate (Doolittle and Booth, 2017). Additionally, we implement the “It's the Song not the Singer” approach proposed by Doolittle (Taxis et al., 2015; Doolittle and Booth, 2017; Doolittle and Inkpen, 2018) by preserving throughout the evolution of the holobiont, those regulatory connections that contribute to the host's adaptation to perform a predefined but otherwise arbitrary dynamical function. The conservation of the dynamical function across generations occurs regardless of the specific host-microbe network interactions that are contributing to the adaptation process.

Our evolutionary model is based on the Boolean network model introduced by S. Kauffman (presented in the M&M section) to describe gene regulation and cell differentiation processes (Kauffman, 1969a,b). During the last 20 years, it has been shown that this model adequately captures the main aspects of gene regulation dynamics. For instance, Boolean networks are able to reproduce gene expression patterns and metabolic pathways experimentally observed in organisms such as *Arabidopsis thaliana* (Espinosa-Soto et al., 2004), *Drosophila melanogaster* (Albert and Othmer, 2003), yeast (Li et al., 2004; Davidich and Bornholdt, 2008), human epithelial cells (Huang et al., 2005) and murine blood progenitor cells (Hameya et al., 2017) among others. Additionally, Huang et al. experimentally showed that the dynamical attractors of a Boolean network correspond to different cell types or cell fates (Huang et al., 2005). Because of this evidence, we use Boolean networks to represent the gene regulation networks of both the hosts and their microbes. Since we are interested in general principles about the emergence of symbiotic interactions, we use random networks instead of carefully constructed ones corresponding to specific organisms. Although the gene regulatory network of an organism greatly determines its phenotype (Davidson and Levine, 2008; Oliveri et al., 2008), it is known that several functions depend more on the general structure of the network than on the specific genes involved (Wagner, 2007). Therefore, using random Boolean networks in our population model has the advantage of determining the capability of the network to acquire new functions throughout its evolution regardless of its detailed composition (Davidson, 2010). This function-centered approach is consistent with the fact that a core microbiome is more likely to be identified based on functionalities rather than on the particular phylogenetic details of its species (Consortium, 2012; Taxis et al., 2015; Doolittle and Booth, 2017; Doolittle and Inkpen, 2018). We describe in detail the Boolean network model in M&M section.

Simulations of this evolutionary model show that the adaptation of the host network is greatly enhanced when it interacts with the microbial networks, which are the ones that absorb most of the mutations without changing their own adaptation. Additionally, the host network can improve its adaptation to perform multiple functions only if the set of microbial networks is partitioned into specialized subsets (niches), each one participating in the host's adaptation to a small number of functions. This specialization provides the holobiont with a structural and dynamical complexity that facilitates its evolution, whereas non-specialized microbiota is shown to be either inconsequential or detrimental to the holobiont's adaptation. Once the holobiont is adapted, the disconnection of one or more of these specialized niches leads a global incompetence to perform the required set of imposed tasks. This is reminiscent of the dysbiosis observed in real organisms when their microbiota's diversity is reduced. To our knowledge, this is the first model in which symbiotic interactions, diversity, specialization and dysbiosis in an ecosystem emerge as a result of coevolution. It also helps us understand the emergence of complex organisms, as they adapt more easily than unstructured ones.

## Model and Results

### Task Assignment

**Figure 1:**
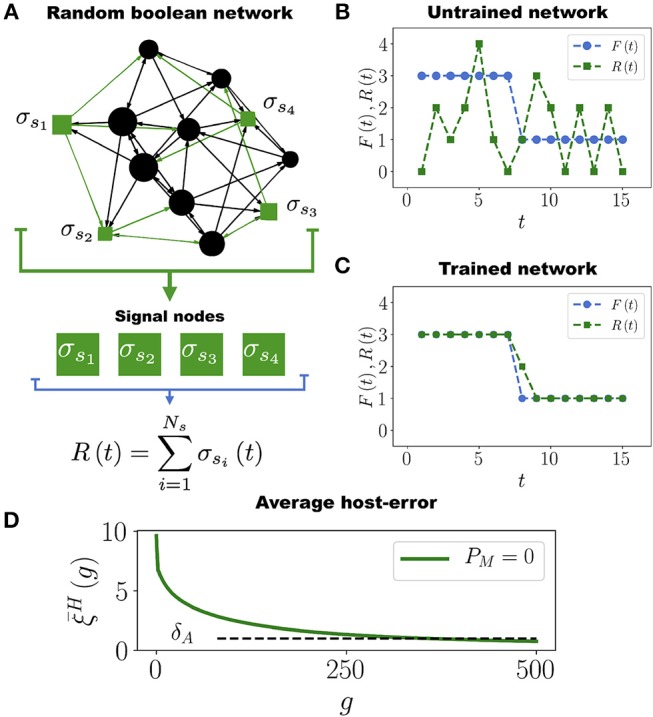
Boolean Network Task. **(A)** Network with *N* = 12 nodes and *N*_*s*_ = 4 signal nodes (represented as green squares), which generate the output signal *R*(*t*) that has to converge to the task *F*(*t*). **(B)** Initially, the untrained network produces an output signal *R*(*t*) (green curve with squares) very different from the target function *F*(*t*) (blue curve with circles). **(C)** After the evolutionary process, the network is well adapted to perform the task. In this example, only one point of the output signal *R*(*t*) acquires a wrong value in the time interval 1 ≤ *t* ≤ 15 in which the task *F*(*t*) is evaluated. **(D)** Average population error ${\bar{\xi }}^{H}$ as a function of the number of generations *g*, for a population with *P* = 100 networks, each having *N* = 50 nodes and *N*_*s*_ = 12 signal nodes. Note that ${\bar{\xi }}^{H}$ decreases and crosses the adaptation threshold δ_*A*_ = 1 approximately at generation *g* = 350, after which most of the networks in the population become well adapted to the task *F*(*t*).

Following the work by Stern (1999), in order to define a task for the Boolean network we start by arbitrarily selecting a subset of *N*_*s*_ nodes that we call *signal nodes*, {σ_*s*_1__, σ_*s*_2__, …, σ_*s*_*N*_*s*___}, from which we extract the *output signal* *R*(*t*) defined as (see Figure 1A)

(1) $$R\left(t\right)=\sum_{i=1}^{N_{s}}\sigma_{s_{i}}\left(t\right).$$

Assigning a task to the Boolean network consists in requiring that the output signal *R*(*t*) approximates as much as possible a predefined *target function* (or *task*) *F*(*t*) (see Figure 1B). In our model *F*(*t*) is an arbitrary function such that 0 < *F*(*t*) < *N*_*s*_ for 1 ≤ *t* ≤ *t*_*m*_, where *t*_*m*_ = 15 is the number of time steps of the assigned task. We set *t*_*m*_ = 15 because this is the average number of time steps it takes for the network to stabilize its dynamics (see Figure S1). In biological terms, the task *F*(*t*) would represent an expression pattern some genes must acquire in order for the organism to efficiently respond to a particular environmental challenge (like yeast responding to a heat shock). Since the networks are randomly constructed, it is expected that initially none of them have this response (their output signal *R*(*t*) and the task *F*(*t*) are usually different at the start of the simulation, see Figure 1B). Therefore, it is necessary to evolve the networks so that *R*(*t*) approaches *F*(*t*) as much as possible, as in Figure 1C. It is only through a series of mutations and adaptations that the phenotype *R*(*t*) will approach *F*(*t*) in some individuals, and then be transmitted to their offspring.

Throughout this work we use networks with *N* = 50 nodes, average connectivity *K* = 2 and *N*_*s*_ = 12 signal nodes (except in some figures where smaller networks are presented for illustrative purposes). The reason for this choice of parameters is the following. It has been observed that genetic networks of several real organisms are structured in functional modules, each one consisting of a few dozen genes or nodes (Resendis-Antonio et al., 2012). For instance, adaptive resistance to antibiotics in *Escherichia coli* is mediated by the MarA-AcrAB-TolC system which, when activated, produces efflux pumps that pump toxic molecules in the intracellular fluid out of the cell, keeping the internal antibiotic concentration below lethal levels. Activation of this system is controlled by a regulatory network consisting of about 15 nodes (Motta et al., 2015). Analogously, the cAMP-dependent protein kinase regulatory network (PKA-RN), which regulates (among other things) the stress response in *Saccharomyces cerevisiae*, consists of 15 nodes (Pérez-Landero et al., 2015). There are many more examples showing that specific cellular functions (such a the response to a given environmental challenge) are controlled by network modules composed of a few dozen nodes (Guo et al., 2016; Ma et al., 2017). Since in this work we are not considering any specific organism, we will assume in a generic way that the task *F*(*t*) that the network has to acquire is encoded in *N*_*s*_ = 12 nodes, which in turn are embedded in a module of *N* = 50 nodes.

Finally, we perform all our simulations using Kauffman networks with connectivity *K* = 2 for two main reasons. First, these networks are trained faster than networks with connectivity smaller or larger than *K* = 2 (see Figure S2). A second, more fundamental reason is that networks with *K* = 2 exhibit critical dynamics, which means that their dynamical behavior is at the brink of a phase transition between order and chaos (Derrida and Pomeau, 1986; Aldana, 2003). Dynamical criticality confers the system interesting properties such as evolvability (i.e., the coexistence of robustness and adaptability) (Aldana et al., 2007; Torres-Sosa et al., 2012), faster information storage, processing and transfer (Langton, 1990; Nykter et al., 2008), and collective response to external stimuli without saturation (Kinouchi and Copelli, 2006), (or shorter training times, as in our case, see Figure S2). There is solid evidence indicating that gene regulatory networks of real organisms are dynamically critical or close to criticality (Shmulevich et al., 2005; Serra et al., 2007; Balleza et al., 2008; Daniels et al., 2018). Therefore, by choosing *K* = 2 we are working with a representative ensemble of networks that have an important dynamical property observed in real organisms.

### Host Network Evolution

We consider a population of *P* = 100 networks, represented as {*H*_1_, *H*_2_, …, *H*_*P*_}, which have to perform the same task *F*(*t*). We will refer to these networks as the *host networks* (HNs). At the start of the simulation all the HNs are identical replicas of one randomly constructed network. To make the output signal of the HNs approach the task *F*(*t*) we implement a traditional evolutionary algorithm in which the networks are mutated, selected and replicated. Variability in the population is implemented by mutating the HNs with a mutation rate μ_*H*_ = 0.001 per node per network per generation. Once a node σ_*n*_ of a given network *H*_*i*_ has been chosen for mutation, we perform any of the following changes with equal probability: (i) Randomly rewire one of the input or output connections of σ_*n*_. (ii) Add a new input (or output) connection to σ_*n*_ from (or to) a randomly chosen node in the network. (iii) Remove one input or output connection of σ_*n*_. (iv) Change one of the entries of the logical function *f*_*n*_ associated to σ_*n*_.

The mutations described above can make each network *H*_*i*_ get closer to the task *F*(*t*) or get away from it. To measure the adaptation of the HNs to the task we denote as *R*_*i*_(*t*) the output signal of the network *H*_*i*_ and define its adaptation error $\xi_{i}^{H}$ as

(2) $$\xi_{i}^{H}={\left(t_{m}\right)}^{-1}\sum_{t=1}^{t_{m}}{\left(R_{i}(t)-F(t)\right)}^{2}.$$

Clearly, if $\xi_{i}^{H}=0$ then the network *H*_*i*_ is perfectly trained (adapted) to perform the task *F*(*t*), whereas large values of $\xi_{i}^{H}$ indicate a poor adaptation. Therefore, when a mutation occurs such that $\xi_{i}^{H}$ decreases, the adaptation of *H*_*i*_ increases and viceversa. We will say that the network *H*_*i*_ is *well adapted* to its task when $\xi_{i}^{H}\leq \delta_{A}$, where δ_*A*_ is the *adaptation threshold*. We set δ_*A*_ = 1, which means that at most one node out of the *N*_*s*_ signal nodes is allowed to deviate one unit from the correct value at every time step during the interval 1 ≤ *t* ≤ *t*_*m*_ over which the task *F*(*t*) is evaluated (see Figure 1C). The average population error, defined as ${\bar{\xi }}^{H}=\frac{1}{P}\sum_{i=1}^{P}\xi_{i}^{H}$, measures the adaptation of the entire population to the task *F*(*t*).

In each generation we mutate the HNs in the population with the mutation rate μ_*H*_. Then, we choose the 10 best networks (those whose errors $\xi_{i}^{H}$ have the lowest values) to get through the next generation while the other 90 networks are removed from the simulation. These 10 networks are replicated by making 9 copies of each one in order to restore the population to its original size *P* = 100. This evolutionary process is repeated until the population crosses the adaptation threshold. A “generation” consists in a full round of mutation, selection and replication processes. Figure 1D shows that the average population error ${\bar{\xi }}^{H}$ decreases throughout generations. This is expected since at each generation we select the networks that minimize the error. From Figure 1D we see that it takes about 350 generations for the average population error of hosts networks to cross δ_*A*_ and become well adapted to the task (see also Movie S1). We have performed simulations with smaller values of the adaptation threshold: δ_*A*_ = 0.5 and δ_*A*_ = 0.2, and the results are qualitatively the same. The only difference is that the smaller the value of δ_*A*_, the longer the computing time for the average population error ${\bar{\xi }}^{H}$ to cross this threshold (see Figure S3). The results presented in Figure 1D correspond to a population of HNs evolving by themselves, i.e., without interacting among them or with other networks. We refer to this case as the *control case*.

### Interaction With the Microbiota: Holobiont Evolution

To model the interaction between the host organism and its microbiota we allow the training of each host network *H* to be assisted by a set of *P*_*M*_ other networks, $B=\left\{M_{1},M_{2},\ldots ,M_{P_{M}}\right\}$, each one representing a *microbial network* (MN). We will refer to the set B as the microbiota, and to the set $L=\left\{H,B\right\}=\left\{H,M_{1},\ldots ,M_{P_{M}}\right\}$, as the holobiont.

Each microbial network $M_{j}\in B$ also has to perform a predefined task $F_{j}^{M}\left(t\right)$, which is an arbitrary function constructed in the same way as the host-network task *F*(*t*). The microbial tasks $F_{1}^{M}\left(t\right),\ldots ,F_{P_{M}}^{M}\left(t\right)$ are different from each other and from *F*(*t*). Before the training of *H* begins, each $M_{j}\in B$ is previously trained to be well adapted to its own task $F_{j}^{M}\left(t\right)$. This means that all the microbial errors $\xi_{j}^{M}$ satisfy, from the very beginning, the well-adapted condition $\xi_{j}^{M}<\delta_{A}$ (as in Figure 1D; the microbial error $\xi_{j}^{M}$ is defined similarly as in Equation (2); see the M&M section for the precise definition). Thus, at generation *g* = 0 the holobiont consists of the untrained host network *H* and a set of well adapted MNs. The evolution of the holobiont then proceeds with the adaptation of *H* to its task and allowing it to interact (as described below) with MNs that already have their own interests. The rationale behind this initial setup is twofold. First, allowing the training of *H* to be assisted by well-adapted MNs captures the fact that at any moment during its evolution, the host organism can recruit from the environment microbial populations already adapted to their environments and able to carry out some functions by their own. Second, we want to determine whether evolutionary conflict emerges between the host and microbial networks when the holobiont evolves as a unit of adaptation, as has been pointed out in Moran and Sloan (2015) and Douglas and Werren (2016). Such a conflict would be apparent in our simulations if a reduction of the host-network error ξ^*H*^ occurs with a simultaneous increase in the average microbial error ${\bar{\xi }}^{M}$, or viceversa.

**Figure 2:**
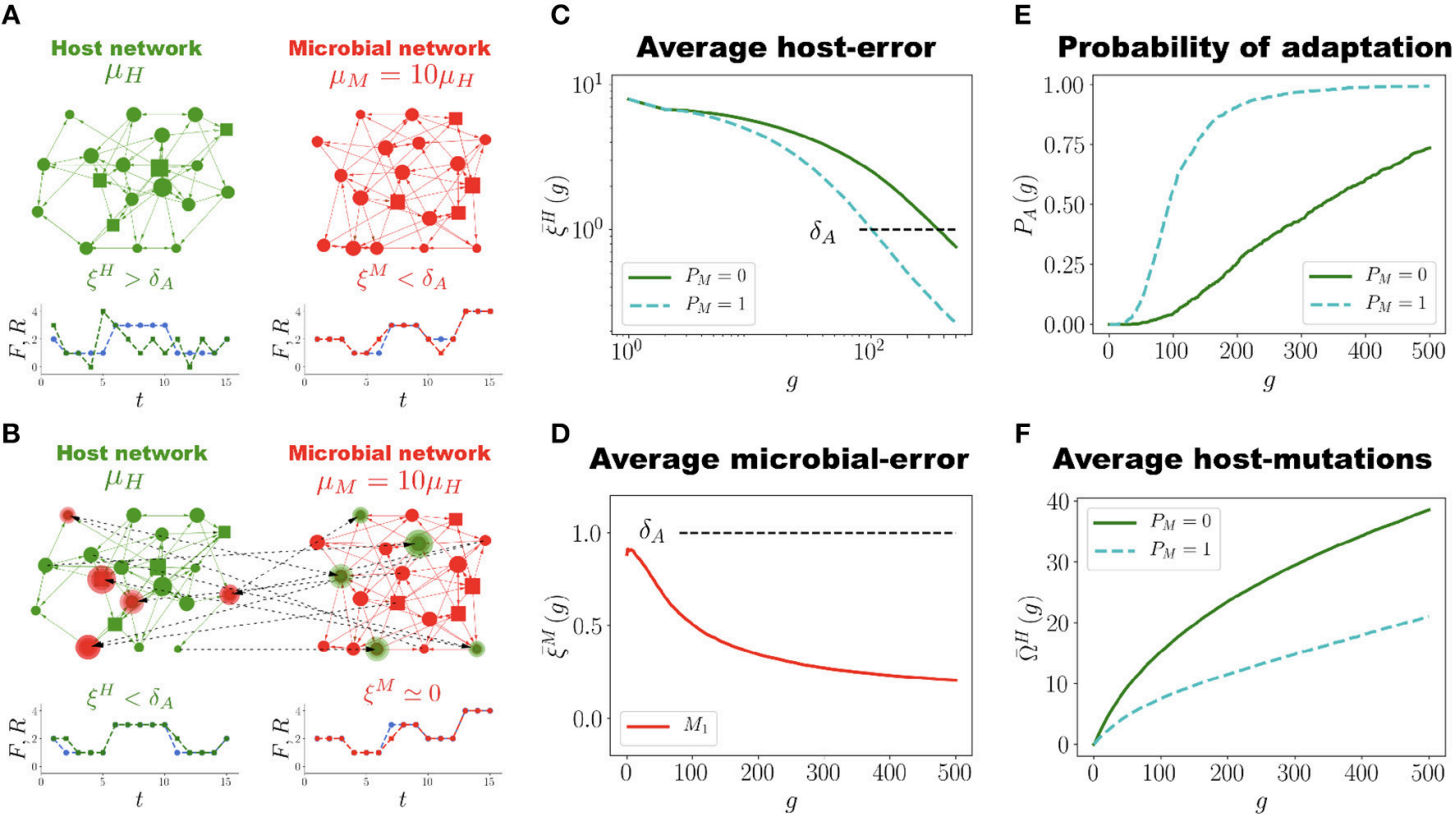
Network Coevolution. **(A)**. Schematic representation of the holobiont, which in this case consists of one host network *H* (green) and one microbial network *M* (red), each with *N* = 20 nodes and *N*_*s*_ = 4 signal nodes (represented by squares). At generation *g* = 0 the host network *H* is not adapted to its task ($\xi^{H}>\delta_{A}$), whereas the microbial network *M* is well adapted ($\xi^{M}<\delta_{A}$). The mutation rates μ_*H*_ and μ_*M*_ of the host and microbial networks, respectively, satisfy μ_*M*_ = 10μ_*H*_. **(B)** At generation *g* = 150 regulatory interactions between *H* and *M* have been established (dashed lines). The highlighted nodes in each network have regulators in the other network. *H* has become well adapted to its task ($\xi^{H}<\delta_{A}$) while the microbial error ξ^*M*^ has decreased almost to zero. **(C)** Population average host error ${\bar{\xi }}^{H}$ as a function of generations for the holobiont case (*H* and *M* evolving together, blue dashed curve, *P*_*M*_ = 1) and for the control case (*H* evolving by itself, green solid curve, *P*_*M*_ = 0). In the holobiont case the adaptability threshold δ_*A*_ is reached faster (*g* ≈ 100) than in the control case (*g* ≈ 350). Also, at the end of the simulation (*g* = 500) the error for the holobiont case is about five times smaller than for the control case. **(D)** Evolution of the average microbial error ${\bar{\xi }}^{M}$. At generation *g* = 0, ${\bar{\xi }}^{M}$ already satisfies the well-adapted condition, ${\bar{\xi }}^{M}<\delta_{A}$, but it further decreases as the evolution of the holobiont goes on. **(E)** Probability of adaptation *P*_*A*_(*g*) across generations for the holobiont (blue dashed curve) and control (green solid curve) cases. Note that *P*_*A*_(*g*) increases and saturates faster in the holobiont case. **(F)** Average number ${\bar{\Omega }}^{H}(g)$ of accumulated mutations in the host network *H* during its adaptation process for the holobiont case (blue dashed curve) and the control case (green solid curve). Interacting with the microbial network halves the number of mutations *H* has to undergo in order to adapt to its task. The numerical simulations to generate the graphs (*C)* to **(F)** were carried out using networks with *N* = 50, *N*_*s*_ = 12 and populations of *P* = 100 networks.

The interaction between *H* and its microbiota B is implemented as follows (see Figures 2A,B). Consider the case where a given node σ_*n*_ of *H* has been chosen for mutation such that a new input (output) connection is to be added. Then this new connection can be selected with equal probability either within *H* itself or from any of the microbial networks $M_{j}\in B$. Likewise, when a given node of a microbial network *M*_*j*_ is mutated so as to receive a new connection (either input or output), the new connection can be established within *M*_*j*_ itself, with *H* or with any other microbial network $M_{k}\in B$. This allows the emergence of regulatory interactions between all the networks that constitute the holobiont.

For the adaptation of *H* to its task we consider the evolution of a population of *P* = 100 holobionts. Throughout the evolutionary process all the networks in each holobiont undergo the same kind of random mutations described in the section Host Network Evolution (with the possibility of interactions across networks, as mentioned in the previous paragraph). However, in our simulations the mutation rate μ_*M*_ for the MNs is ten times larger than the mutation rate μ_*H*_ for the host network, namely μ_*M*_ = 10μ_*H*_. This captures the fact that bacterial colonies, due to their high reproduction rates, develop mutants at least ten times faster than populations of eukaryotic cells in multicellular organisms (Lynch, 2010; Lynch et al., 2016). It is important to emphasize that in our model each network in the holobiont has to be considered not as representing a single cell, but an entire cell population. In each generation, holobionts are ranked according to their error ξ^*L*^, and the ten with the smallest errors are selected for reproduction (see M&M). In all further simulations the unit of selection is the holobiont, as in each generation we select the ten best holobionts (based on the error ξ^*L*^ that takes into account the host and microbial errors) and replicate them.

Figure 2C shows the average population error ${\bar{\xi }}^{H}$ of the host network *H* across generations for holobionts as well as for the control case (host networks evolving by themselves without interacting with microbial networks). In the simulations reported in Figure 2C each holobiont consists of one host network *H* and one microbial network *M* (*P*_*M*_ = 1). It is clear that interacting with only one microbial network *M* already makes *H* to adapt much faster to its task than evolving on its own. In the holobiont case, the error ${\bar{\xi }}^{H}$ crosses the adaptability threshold δ_*A*_ in about one fourth of the generations required for the control case to do it (see Movie S2 and compare with Movie S1). Furthermore, the final error after 500 generations is considerably smaller for the holobiont case (${\bar{\xi }}^{H}\approx 0.2$) than for the control case (${\bar{\xi }}^{H}\approx 0.95$). Note that an error ${\bar{\xi }}^{H}\approx 0.2$ means that, on average, at most 3 points of the output signal *R*(*t*) deviate one unit from the task *F*(*t*) in the whole interval 1 ≤ *t* ≤ 15, which represents a percent error 100 × 3/(*N*_*s*_×15) ≈ 1.6%. This is almost a perfect adaptation hard to achieve in the control case. For the control case, it takes about 3000 generations to reach a similar error of ${\bar{\xi }}^{H}\approx 0.2$ (See Figure S3). The average microbial error ${\bar{\xi }}^{M}$ also decreases, as shown in Figure 2D. At generation *g* = 0, ${\bar{\xi }}^{M}$ is already below the adaptability threshold δ_*A*_, but it decreases even further as the evolution of holobionts proceeds. Therefore, the adaptation of the holobiont takes place with no conflict of interest between *H* and its microbial networks.

In Figure 2E we report the probability of adaptation *P*_*A*_(*g*), defined as the fraction of holobionts in which the host-network error ξ^*H*^ crosses the adaptation threshold δ_*A*_ at generation *g*. It is apparent from Figure 2E that this probability for the holobiont case increases and saturates much faster than for the control case. About 80% of the holobionts are well adapted after only 120 generations, whereas host networks evolving by themselves never reach 75% of adaptation during the whole simulation time. In addition to speeding up and increasing the adaptation of *H*, the interaction between *H* and *M* also considerably reduces the number of mutations *H* has to accumulate in order to adapt to its task, as Figure 2F reveals. This is not a trivial result, for only the mutations in both *H* and *M* that increase the adaptation of the holobiont are selected and fixed in the population. Thus, even though *M* mutates ten times faster than *H*, not all of those mutations are beneficial to the adaptation of the holobiont and consequently, not all of them become fixed in the population. Actually, from Figure 2E we observe that the average number ${\bar{\Omega }}^{H}$ of accumulated mutations in *H* to reach the adaptation threshold δ_*A*_ is not ten, but only two times larger for the control case than for the holobiont case. However, it is true that because μ_*M*_ is larger than μ_*H*_ the adaptation of *H* is improved (our simulations show that there is no significant difference between the holobiont and control cases when μ_*M*_ = μ_*H*_, see Figure S4).

### Symbiosis and Dysbiosis

**Figure 3:**
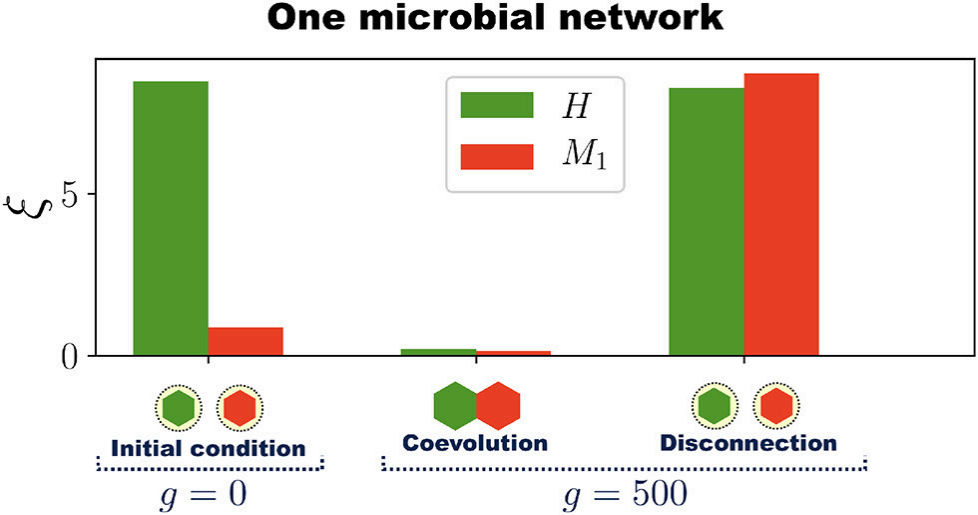
Emergence of symbiosis and dysbiosis. A holobiont consisting of one host network *H* and one microbial network *M*_1_ evolves for 500 generations. Then *H* and *M*_1_ are disconnected and the errors at performing their respective tasks evaluated (*H* and *M*_1_ are represented by hexagons at the bottom of the bar chart). At generation *g* = 0, when the training of *H* begins, the host-network error ξ^*H*^ (green bar) is large whereas the microbial-network error ξ^*M*^ (red bar) is already below the adaptation threshold δ_*A*_. After *H* and *M*_1_ have coevolved for 500 generations both ξ^*H*^ and ξ^*M*^ are quite below δ_*A*_, which indicates the adaptation of the entire holobiont. Then, *H* and *M*_1_ are disconnected and their respective errors evaluated. Note that after disconnection both errors ξ^*H*^ and ξ^*M*^ in this example increase to levels corresponding to completely untrained networks. The simulations were performed with networks having *N* = 50 nodes and *N*_*s*_ = 12 signal nodes.

To show that symbiotic relationships emerge between the host and microbial networks, once the holobiont is well adapted (after 500 generations as in Figure 2C,D), we remove the connections between *H* and *M* (the dashed lines in Figure 2B) and compute the errors of each network at performing their respective tasks while disconnected. This can be thought of as an antibiotic administration where several bacterial species are removed from the microbial population, or as trying to cultivate these symbiotic microbes without their respective host. Thus, a set of microbial species, represented by *M*, are removed from the holobiont and then the fitness of the host is evaluated without them. At the same time, we determine the survivability of these microbial species *M* in the absence of their host. Since *M* starts the evolutionary process already well adapted to its task, one can expect that its error does not significantly increase after the connections between *H* and *M* are removed. However, Figure 3 shows a typical example in which removing the connections between *H* and *M* increases both errors ξ^*H*^ and ξ^*M*^ to values that correspond to untrained networks. Thus, in the example shown in Figure 3, after the holobiont has been adapted as a whole, none of the networks it consists of can perform their respective tasks when separated (see Figure S5 for population statistical averages).

### Multitasking and Microbial Diversity

So far we have presented results in which the host network *H* has to perform only one task. Interaction with one MN significantly improves the adaptation of *H* (and of the holobiont) and reduces the number of mutations it has to undergo in order to become well adapted. It could be expected that adding more MNs to the microbiota would further enhance the adaptation of *H*. However, this is not the case. Adding more MNs either has no effect or can even worsen the adaptation of the host (see Movie S3 and Figure S6). This result is in contradiction with the great diversity observed in the microbiota of real organisms and the ability of the holobiont to adequately perform multiple tasks.

For this reason, we now consider the case in which the host network *H* is trained to perform *T* multiple tasks *F*_1_(*t*), *F*_2_(*t*), …, *F*_*T*_(*t*), each being an arbitrary function constructed as described in the Task Assignment section. Since Boolean Network dynamics are deterministic, depending on the initial condition the dynamics of *H* will be set to follow a specific task *F*_τ_(*t*). We can measure the adaptation errors $\xi_{\tau }^{H}$ and $\xi_{j,\tau }^{M}$ of *H* and the microbial network $M_{j}\in B$, respectively, when *H* is being trained to perform the particular task *F*_τ_(*t*) (see the M&M section for a precise definition of $\xi_{\tau }^{H}$ and $\xi_{j,\tau }^{M}$). This allows us to compute the adaptation of the holobiont separately for each task. Averaging $\xi_{\tau }^{H}$ and $\xi_{j,\tau }^{M}$ over all the tasks gives us the total adaptation errors ξ^*H*^ and $\xi_{j}^{M}$ for the host and microbial networks, respectively (see the M&M section).

**Figure 4:**
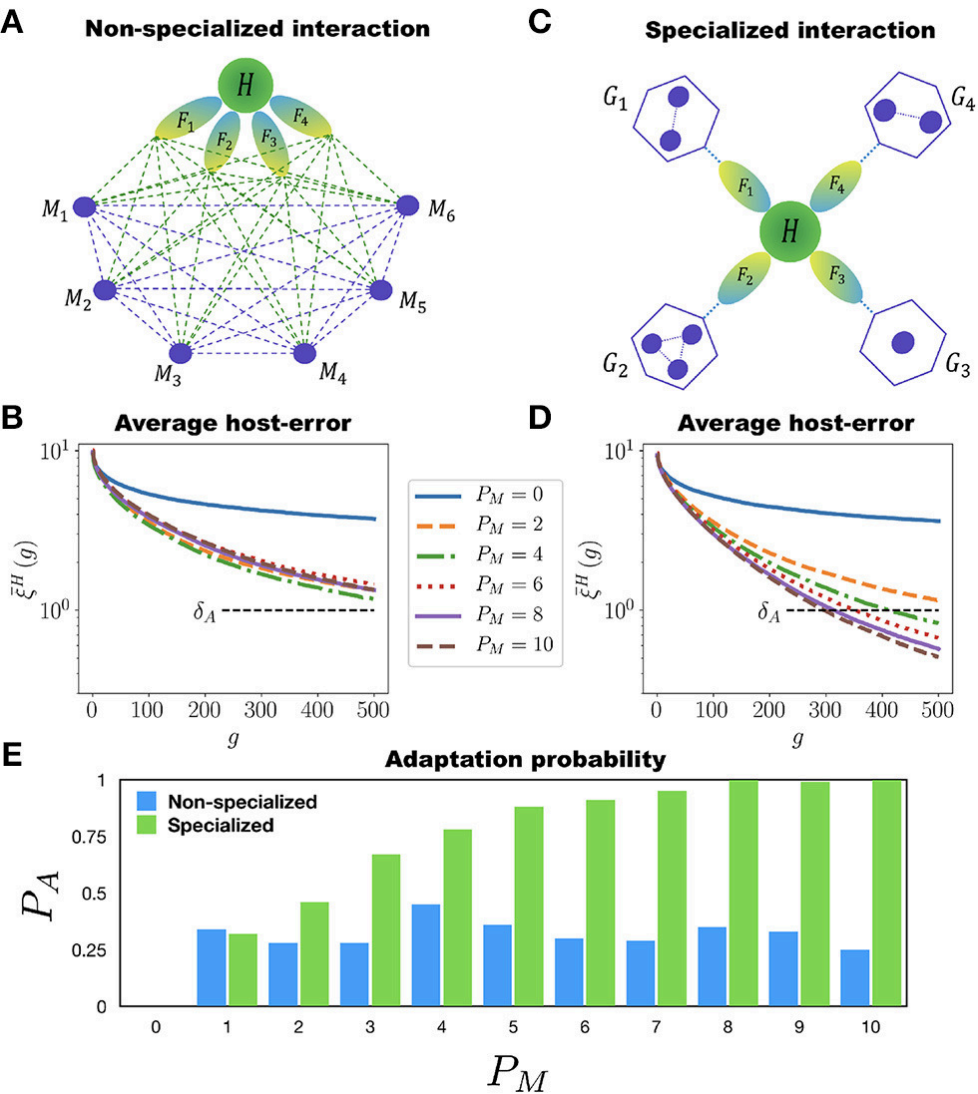
Multitasking and microbial specialization. The host network *H* (green circle) has to perform *T* different tasks *F*_1_, *F*_2_, …, *F*_*T*_ (represented as ellipses) assisted by *P*_*M*_ microbial networks *M*_1_, *M*_2_, …, *M*_*P*_*M*__ (small circles). **(A)** Schematic representation of a holobiont with non-specialized interaction in the microbial networks. Each microbial network can interact with all the other ones and participate in the adaptation of *H* to all of its tasks. The dashed lines represent possible interactions. **(B)** Evolution of the host-network error ${\bar{\xi }}^{H}$ in the non-specialized case for *T* = 10. Each curve corresponds to a different value of *P*_*M*_, starting from *P*_*M*_ = 0 (*H* evolving by itself) up to *P*_*M*_ = 10. In none of these cases ${\bar{\xi }}^{H}$ crosses the adaptation threshold δ_*A*_ even after 500 generations. Furthermore, adding more than one microbial network to the holobiont has a negligible effect on the adaptation of *H*. **(C)** Schematic representation of the holobiont with specialized interaction in the microbial networks. The set of *P*_*M*_ microbial networks is divided into *T* different niches, *G*_1_, …, *G*_*T*_, each one participating in the adaptation of *H* to only one task. The microbial networks can interact among them only if they belong to the same niche. **(D)** Host-network error ${\bar{\xi }}^{H}$ across generations for the specialized interaction and *T* = 10. Again, each curve corresponds to a different number *P*_*M*_ of microbial networks. Note that in this case the more microbial networks in the holobiont the better the adaptation of *H*. Note also that for *P*_*M*_ = 10 the host-network error ${\bar{\xi }}^{H}$ crosses the adaptation threshold δ_*A*_ in less than 300 generations. **(E)** Adaptation probability *P*_*A*_ at generation *g* = 500 as a function of the number *P*_*M*_ of microbial networks participating in the host's adaptation. The blue and green bars correspond to the non-specialized and specialized cases, respectively. Note that in the non-specialized case the adaptation probability remains low regardless of the number of microbial networks, whereas in the specialized case, the more microbial networks the better the adaptation of the host. The simulations were carried out for populations of *P* = 100 holobionts and networks with *N* = 50 nodes.

We implement two ways in which the MNs can assist the adaptation of *H* to perform many different tasks. First, there is the *non-specialized interaction* case in which all the MNs can interact among each other and with *H*. Also, all the MNs can participate in the adaptation of *H* to all of its tasks (see Figure 4A). In each generation the networks are mutated, allowing new interactions to appear between any two networks within the holobiont. This means that new incoming or outgoing connections can be established either between *H* and any of its MNs, or between any two MNs. We consider again a population of *P* = 100 holobionts. After the networks in each holobiont have been mutated (with the mutation rates μ_*H*_ and μ_*M*_ for the host and microbial networks, respectively), the ten best holobionts are selected and replicated (see the M&M section for a definition of the holobiont error ξ^*L*^ in the multitasking case). Figure 4B shows the population average ${\bar{\xi }}^{H}$ of the host-network error for the case in which *H* has to perform 10 different tasks. It is clear from this figure that adding more than one microbial network to the microbiota has no effect on the adaptation of *H* to its 10 different tasks. Therefore, in the non-specialized case increasing the diversity of the microbiota does not help the adaptation rate of the host.

As a second alternative we implement a *specialized interaction* in the microbial networks. In this case the microbiota $B=\left\{M_{1},\ldots ,M_{P_{M}}\right\}$ is divided into *P*_*G*_ disjoint non-empty subsets, or “niches”, {*G*_1_, *G*_2_, …, *G*_*P*_*G*__}. The set of tasks $F=\left\{F_{1},\ldots ,F_{T}\right\}$ is also partitioned, as evenly as possible, into *P*_*G*_ non-overlapping subsets, $\left\{T_{1},T_{2},\ldots ,T_{P_{G}}\right\}$. The maximum number of niches is *P*_*G*_ = *T*, for in this case each subset $T_{\tau }$ contains only one task. To each niche *G*_τ_ we associate a subset $T_{\tau }$ of tasks (see Figure 4C). The host network *H* is still trained to perform the *T* different tasks *F*_1_, …, *F*_*T*_. However, the training of *H* to the tasks in the particular set $T_{\tau }$ is assisted only by the networks in the corresponding niche *G*_τ_. For each niche *G*_τ_ we compute an error $\xi_{\tau }^{G}$ that measures the adaptation of the holobiont when *H* is being trained to perform the tasks in the specific subset $T_{\tau }$ (see the M&M section for a precise definition of the niche error $\xi_{\tau }^{G}$). During the adaptation of *H* to the tasks in $T_{\tau }$, only the mutations in *H* or in the microbial networks belonging to *G*_τ_ that reduce the corresponding error $\xi_{\tau }^{G}$ are selected. The important point to note here is that the adaptation of *H* to its tasks can be measured separately for each niche. The holobiont error ξ^*L*^ is computed as the average of the errors $\xi_{\tau }^{G}$ over all the niches (see the M&M section).

During the training of *H*, the MNs in one niche can develop interactions between them, but they cannot interact with the MNs in a different niche, as Figure 4C indicates. This is consistent with the observation that multicellular organisms maintain the stability of its microbiota by reducing microbial interactions (Deines et al., 2017). If microbes interacted with no organization, the loss of one microbial species would affect the fitness of all the others, increasing the risk of extinction cascades (Coyte et al., 2015). Therefore, in the specialized interaction case we compartmentalize the MNs allowing interactions among them only if they belong to the same niche (all the MNs in all the niches can, of course, interact with the host network *H*).

Figure 4D shows the evolution of the average host-error ${\bar{\xi }}^{H}$ for simple case where each niche has one MN (*P*_*G*_ ≤ *T*). It is clear from Figure 4D that, contrary to the non-specialized case, adding more MNs to the microbiota in a specialized way considerably improves the adaptation of *H* to its multiple tasks. Furthermore, in Figure 4E we report the probability of adaptation *P*_*A*_(500) at generation *g* = 500 as a function of *P*_*M*_ for both the non-specialized and specialized cases. In the former case *P*_*A*_ remains low and never improves as *P*_*M*_ increases, whereas in the later case *P*_*A*_ monotonously grows with *P*_*M*_. This clearly shows that both diversity and specialization of the MNs are necessary for *H* to adapt to multiple tasks.

**Figure 5:**
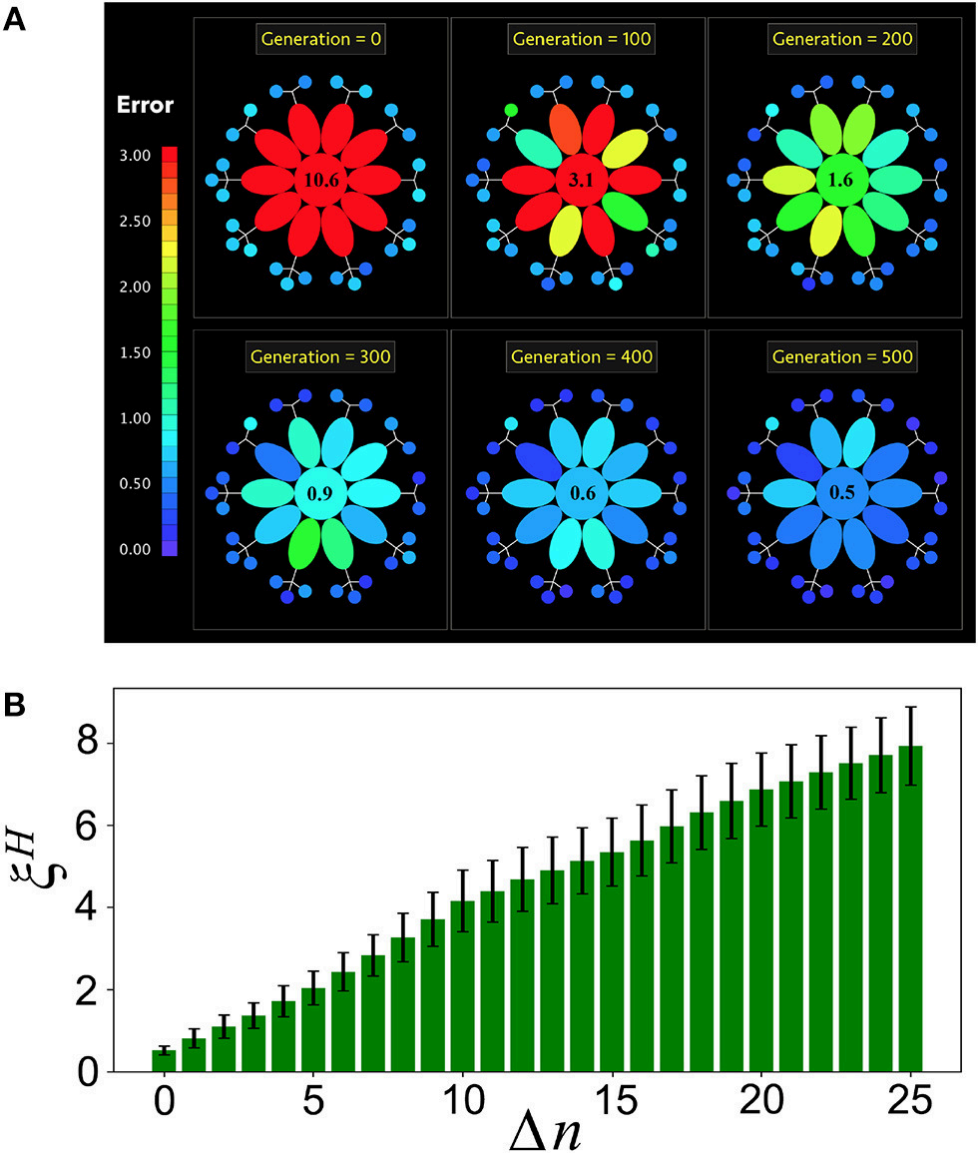
Holobiont's complexity and loss of microbial diversity. **(A)** Snapshots of the evolution of a holobiont in the specialized case. The holobiont consists of *T* = 10 tasks (ellipses) and *P*_*M*_ = 25 microbial networks (small circles). All errors larger than 3 are colored in red. At generation *g* = 0 the host network *H* has a large error in all of its tasks, while the bacterial networks are well adapted. As the evolution proceeds, the error of the entire holobiont decreases, but the adaptation of *H* to its different tasks takes place at different rates. The number at the center is the value of the host error ${\bar{\xi }}^{H}$ averaged over all of its tasks. **(B)** After the holobiont is well adapted at generation *g* = 500, Δ*n* microbial networks are disconnected and the resulting host-network error ${\bar{\xi }}^{H}$ is computed. This simulates the effect on the holobiont's adaptation of a reduced microbial diversity. Note that ${\bar{\xi }}^{H}$ gradually increases as more microbial networks are disconnected, indicating that the host becomes less adapted as the microbial diversity decreases.

The results presented for the specialized interaction case also hold when every niche is populated with more than one MN (*P*_*M*_ ≥ 2*T*). In Figure 5A (see also Movie S4) we report the evolution of a holobiont with *T* = 10 different tasks and the same number of niches, and *P*_*M*_ = 25 microbial networks (each niche contains either two or three MNs). At generation *g* = 0, *H* is poorly adapted to all of its tasks (represented in red), whereas all the MNs are already well adapted (represented in blue). As the evolution proceeds *H* becomes more adapted to all of its tasks. Furthermore, the microbial networks also become more adapted to their own tasks. Note that the adaptation of *H* to its different tasks occurs at different rates, as can be seen from the color-code of the tasks through the holobiont evolution. This is consistent with the observation that different symbiotic relationships between the host and its microbial communities emerge at different rates (Doolittle and Booth, 2017). In the example shown in Figure 5A the adaptation of the whole holobiont crosses the adaptation threshold δ_*A*_ in <300 generations. Interestingly, the same results are obtained in the specialized case when many more microbial networks are introduced into the holobiont (see Figure S7).

The specialized interaction scheme allows us to compute the robustness of the holobiont under loss of microbial diversity. For this, once the holobiont is well adapted, we disconnect Δ*n* microbial networks from it and compute the resulting host-network error ξ^*H*^ averaged over all the host's tasks. Figure 5B shows that ξ^*H*^ gradually increases as more microbial networks are disconnected from the holobiont. Therefore, a loss in microbial diversity clearly reduces the adaptation of the host.

## Discussion

Multicellular organisms and microbes have coevolved in many different ways, not only as holobionts being units of adaptation (Theis et al., 2016). However, the persistence across generations of regulatory interactions between the host and its microbes is a necessary condition for natural selection to operate at the holobiont level (Doolittle and Booth, 2017). These regulatory interactions have to preserve the holobiont's functionality regardless of the specific microbial species that generate them. This is the “It's the song not the singer” (ITSNTS) approach to evolution proposed by Doolittle (Doolittle and Booth, 2017; Doolittle and Inkpen, 2018) and exemplified by Taxis et al. in ruminal ecosystems (Taxis et al., 2015). In this work we have incorporated into a single evolutionary model both the concept that the holobiont is a unit of selection and Doolittle's ITSNTS approach. We have done so by requiring in our simulations that only the best adapted holobionts at each generation are the ones able to go throughout the selective filter, pass to the next generation and replicate all of its constituent networks. However, in our model selection acts on a dynamical property of the holobiont, which is the host's output signal, in order to bring it close to the functions the host needs to perform. In this scheme, it does not matter what nodes or microbial networks participate in the regulation of the dynamical functions. What is important is the preservation across generations of the dynamical functions themselves, and this must hold for both the host and the microbial networks (which also have to perform and preserve their own functions). Thus, although the holobiont might not be the only unit of selection (Theis et al., 2016), we have concentrated on those important host-microbe co-interactions that transmit functionality across multiple generations (Roughgarden et al., 2018). Our model does not assume that the microbial networks reside inside the host, but only that they interact with it and that the host-microbe interactions are transmitted across generations. This propagation can happen in various ways other than vertical transmission from parents to offspring as, for instance, when the host constructs its environment with a stable microbial composition (Fitzpatrick, 2014).

We have shown that the host network can actually be trained to perform one task without the help of any microbial networks, as Figure 1D and Figure S3 illustrate. However, allowing interactions between the host and microbial networks greatly speeds up and improves the adaptation of the entire holobiont. This is because the host network does not only adapt to its tasks faster, better and with less mutations when it is allowed to interact with microbial networks, but the microbial networks themselves considerably improve their own adaptation to their respective tasks. Furthermore, adaptation of the host network to perform *multiple* tasks is improved only when it is allowed to interact with a diverse and specialized microbiota, as Figure 4D shows.

In light of these results, we observe that the microbiota does not only help the host to adapt to its tasks. There is mutual benefit in which both the host and its microbial communities contribute to each other's adaptation. It is in this sense that the holobiont can be considered as an evolutionary unit.

It is important to mention that in our model the holobiont cannot just be considered as one “big network” evolving to perform a set of tasks. There are two essential aspects that have to be emphasized. First, the rate at which mutants are generated μ_*M*_ in the microbial networks is considerably larger than that μ_*H*_ of the host networks. Second, the set of microbial networks must be partitioned into disjoint (i.e., non-interacting) niches for the host network to efficiently adapt to multiple tasks, where each niche specializes in the adaptation of the host to one specific subset of tasks. These two aspects provide the holobiont with a complex internal dynamical structure that prevents us from viewing it as just one big network (see Figure 5A and Figure S7). A holobiont for which μ_*M*_ = μ_*H*_ and the microbial networks are not partitioned into specialized niches, could be considered as a single large homogeneous network. But in such a homogeneous case the holobiont evolution benefits neither from the host-microbe interactions nor from the microbiota's diversity (see Figures S4, S8). Rather, the structural and dynamical internal complexity of the holobiont, embodied in the functional modularity and specialization of the microbial niches as well as in the difference between the host and microbial mutation rates, is required to facilitate and improve the holobiont's adaptation to perform multiple tasks. Hence, our results show that complexity, modularity and functional specialization are necessary properties that naturally facilitate the evolution and adaptation of the holobiont as well as the diversification of the microbiota (Sachs et al., 2014), whereas structural and functional homogeneity is either inconsequential or even detrimental to the holobiont's evolution.

One may wonder whether the relationship μ_*M*_ = 10μ_*H*_ between the microbial and host network's mutant-generating rates accurately reflects reality. We have explored a wide range of values of the ratio γ = μ_*M*_/μ_*H*_, ranging from γ = 1 to γ = ∞. The latter case corresponds to μ_*H*_ = 0, which means that the adaptation of the host to its task does not occur across generations, but within the host's lifespan. In this extreme case, the adaptation of the host to its task occurs due to mutations in the microbiota but not in the host itself. Our simulations show that the adaptation of the host network is almost equally accelerated and improved for γ = 10 than for γ = ∞ (see Figure S9).

In our model the host network interacts with microbial networks which, from the very beginning, are already well adapted to perform their own functions. The reason for this is to determine whether or not the well-adapted condition imposed on the microbial networks represents a restriction that could generate evolutionary conflict within the holobiont. It has been pointed out that the emergence of symbiotic relationships between organisms requires the symbionts to be highly cooperative and show very little conflict (Morris et al., 2012; Sachs and Hollowell, 2012; Sachs et al., 2014; Queller and Strassmann, 2016). Our simulations show that the evolution of the holobiont can very well take place with no evolutionary conflict between its constituent networks, as long as the microbiota is partitioned into specialized niches. This modularization and division of labor are essential to prevent microbial competition and within-group conflict in the holobiont (West et al., 2015) (see Figure S6). Additionally, modularization of the microbiota allows the holobiont to acquire new functions without affecting the ones already present.

Interestingly, similar results regarding the adaptation of the host network to its tasks are obtained when the well-adapted condition is not imposed on the microbial networks. Our simulations show that the adaptation of the host network is equally accelerated and improved when it interacts with microbial networks that do not have to perform any task (see Movie S5). However, even when the microbial networks are free of any selective pressure, their dynamics are stabilized when they coevolve with the host network (see Movie S5). This is important because it can be interpreted as the holobiont acquiring, at any moment, microbes from the environment and coevolving with them, generating intergenomic epistasis that reduces within-group conflict and promotes the adaptation of the entire holobiont (Bordenstein and Theis, 2015).

Finally, we would like to mention that, although there exist many qualitative taxonomical studies showing the existence of a great variety of host-microbe symbiotic interactions, there are very few mathematical and computational models aiming to explain the general mechanisms responsible for the emergence of such interactions and the need for diversification and specialization of the microbiota (Manor et al., 2014). We have not explicitly considered competition or parasitism in our model. However, by integrating the ITSNTS approach with the hologenome hypothesis (the holobiont as a unit of selection Rosenberg and Zilber-Rosenberg, 2016; Roughgarden et al., 2018), we were able to reproduce many of the observed behaviors in the evolution of holobionts, such as reduction of evolutionary conflict, division of labor, emergence of symbiotic interactions and dysbiosis when the microbiota diversity is reduced. Our model may thus lay the foundations for a comprehensive understanding of the long-lasting coevolution of multicellular organisms and microbes.

## Materials and Methods

### Boolean Network Model

The Boolean network consists of a set of *N* nodes {σ_1_, σ_2_, …, σ_*N*_}, each acquiring the values 0 or 1 that represent two possible states of activity: “active” or “inactive.” The value of each node σ_*n*_ is determined through a logical function *f*_*n*_ that depends on a set of *k*_*n*_ other nodes in the network denoted as $I_{n}=\left\{\sigma_{1}^{n},\sigma_{2}^{n},\cdots \;,\sigma_{k_{n}}^{n}\right\}$. The nodes in the set *I*_*n*_ are known as the *inputs* or *regulators* of σ_*n*_. In the context of genetic networks these regulators together with the logical function *f*_*n*_ mimic the effect of *k*_*n*_ transcription factors (synthesized by the regulators) acting on the expression of σ_*n*_. For networks of real organisms both the logical function *f*_*n*_ and the set of regulators *I*_*n*_ associated to each gene are carefully constructed according to the activating and inhibitory nature of the regulatory interactions between the genes. Here, the *k*_*n*_ regulators of each node σ_*n*_ are randomly chosen from anywhere in the network. The logical functions *f*_*n*_ are also randomly chosen from the ensemble of all possible logical functions with *k*_*n*_ variables. In this work we start the simulations with networks for which *k*_*n*_ = *K* = 2, which means that every node in the initial networks has exactly *K* = 2 regulators (chosen randomly). Note that this is a directed network because if node σ_*m*_ is a regulator of σ_*n*_, it does not necessarily happen that σ_*n*_ is also a regulator of σ_*m*_ (although it may happen). Note also that throughout the evolution of the networks some input and output connections are added to, or removed from, different nodes. Therefore, the final networks do not have a constant connectivity *K* = 2 for every node. The final networks will have a connectivity distribution similar to the one observed in the Erdös-Rényi topology with an average around *K* = 2 (see Movies S1, S2). Once each node in the network has been provided with a set of inputs and a logical function, the network dynamics is given by

(3) $$\sigma_{n}\left(t+1\right)=f_{n}\left(\sigma_{1}^{n}\left(t\right),\sigma_{2}^{n}\left(t\right),\cdots ,\sigma_{k_{n}}^{n}\left(t\right)\right).$$

Starting the dynamics from an initial condition Σ = {σ_1_(0), σ_2_(0), …, σ_*N*_(0)}, the network transits throughout a series of states until a periodic pattern is reached, which is known as a *dynamical attractor*. There is a great body of work showing that the dynamical attractors of the network correspond to different cell types or cell fates (or more generally, to different functional states of the cell). Here we are not interested in the dynamical attractors, but in training the network to perform a specific task.

### Microbial and Holobiont Errors: One Task

Let us consider a holobiont $L=\left\{H,M_{1},\ldots ,M_{P_{M}}\right\}$. When the host network *H* has to perform only one task *F*(*t*), its error ξ^*H*^ is given in Equation (2). We similarly define the microbial error $\xi_{j}^{M}$ corresponding to the *j*^*th*^ microbial network *M*_*j*_ as $\xi_{j}^{M}=\frac{1}{t_{m}}\sum_{t=1}^{t_{m}}{\left(R_{j}^{M}(t)-F_{j}^{M}(t)\right)}^{2}$, where $R_{j}^{M}\left(t\right)$ and $F_{j}^{M}\left(t\right)$ are the output signal and target function of *M*_*j*_, respectively. Different tasks are assigned to the different microbial networks. The error ξ^*L*^ of the entire holobiont is then computed as

(4) $$\xi^{L}=\frac{1}{1+P_{M}}\left(\xi^{H}+\sum_{j=1}^{P_{M}}\xi_{j}^{M}\right).$$

In our simulations the population contains *P* = 100 holobionts, $L_{1},L_{2},\ldots ,L_{P}$. For each holobiont $L_{i}$ we compute its error $\xi_{i}^{L}$ as in Equation (4), which is then used at each generation to select the best holobionts in the population.

We also preformed numerical simulations using the following definition for the holobiont error:

(5) $$\xi^{L}=\frac{1}{2}\left(\xi^{H}+\frac{1}{P_{M}}\sum_{j=1}^{P_{M}}\xi_{j}^{M}\right).$$

The difference between Equations (4 and 5) is the contribution of the microbiota to the holobiont's error. In Equation (4) the contribution of the total microbial error could be very large as compared to the contribution of the host-network error, especially if there are many microbial networks in the holobiont. Contrary to this, in Equation (5) the microbiota and the host have the same contribution regardless of the number of microbial networks in the microbiota. Our simulations show that both definitions produce qualitatively the same results (see Figure S6B). This is because at each generation in the evolutionary process we are selecting the best holobionts in the population, and selecting the best holobionts eventually leads to the same type of individuals regardless of the way in which the contribution of each particular network is weighted. In this work we present results using the definition given in Equation (5).

### Host and Microbial Errors: Multitasking Non-specialized Case

Let us consider a holobiont $L=\left\{H,M_{1},\ldots ,M_{P_{M}}\right\}$ where now the host network *H* has to perform *T* different tasks *F*_1_(*t*), *F*_2_(*t*), …, *F*_*T*_(*t*). For each task *F*_τ_(*t*) the network starts its dynamics from a predefined initial condition $\Sigma_{\tau }=\left\{\sigma_{1}^{\tau }\left(0\right),\sigma_{2}^{\tau }\left(0\right),\ldots ,\sigma_{N}^{\tau }\left(0\right)\right\}$. Let us denote as *R*_τ_(*t*) the output signal of *H* when it starts its dynamics from the initial condition Σ_τ_. The error $\xi_{\tau }^{H}$ corresponding to the task *F*_τ_(*t*) is computed as

(6) $$\xi_{\tau }^{H}=\frac{1}{t_{m}}\sum_{t=1}^{t_{m}}{\left(R_{\tau }(t)-F_{\tau }(t)\right)}^{2}.$$

This allows us to measure the adaptation of the host network to each of its tasks separately. The total adaptation error ξ^*H*^ of *H* is computed by averaging the corresponding errors over all the *T* tasks that *H* has to perform: $\xi^{H}=T^{-1}\sum_{\tau =1}^{T}\xi_{\tau }^{H}$.

To define the error $\xi_{j,\tau }^{M}$ of the microbial network *M*_*j*_ when the host network is being trained to perform the task *F*_τ_(*t*) we have to consider the fact that *H* may be regulating some of the nodes of *M*_*j*_ (throughout the evolution of the holobiont such regulations may appear). Therefore, the output signal of *M*_*j*_ depends on the initial condition Σ_τ_ used to start the dynamics of *H*. Let us denote as $R_{j,\tau }^{M}$ the output signal of *M*_*j*_ when *H* started its dynamics from the initial condition Σ_τ_. The corresponding microbial error $\xi_{j,\tau }^{M}$ is then defined as

(7) $$\xi_{j,\tau }^{M}=\frac{1}{t_{m}}\sum_{t=1}^{t_{m}}{\left(R_{j,\tau }^{M}(t)-F_{j}^{M}(t)\right)}^{2},$$

where $F_{j}^{M}$ is the task assigned to the microbial network *M*_*j*_ (this network was already well adapted to its tasks and has to remain so during the evolutionary process). The total microbial error $\xi_{j}^{M}$ corresponding to *M*_*j*_ is then computed by averaging $\xi_{j,\tau }^{M}$ over all the tasks: $\xi_{j}^{M}=T^{-1}\sum_{\tau =1}^{T}\xi_{j,\tau }^{M}$. The holobiont error ξ^*L*^ is computed using Equation (4), where now ξ^*H*^ and $\xi_{j}^{M}$ are the host and microbial errors averaged over all the tasks as described above.

### Niche Error: Multitasking Specialized Case

Let us consider the niche *G*_τ_ = {*M*_τ_1__, *M*_τ_2__, …, *M*_τ_*p*_τ___}, containing *p*_τ_ microbial networks. This niche is helping *H* to adapt to the tasks in the set $T_{\tau }=\left\{F_{\tau_{1}},F_{\tau_{2}},\ldots ,F_{\tau_{q_{\tau }}}\right\}$, which contains *q*_τ_ tasks. The error $\xi_{\tau }^{G}$ corresponding to this niche is defined as

(8) $$\xi_{\tau }^{G}=\frac{1}{1+p_{\tau }}\left(\xi_{\tau }^{H}+\sum_{i=1}^{p_{\tau }}\frac{1}{q_{\tau }}\sum_{j=1}^{q_{\tau }}\xi_{\tau_{i},\tau_{j}}^{M}\right),$$

where $\xi_{\tau }^{H}$ and $\xi_{\tau_{i},\tau_{j}}^{M}$ are defined in Equations (6, 7), respectively (in the latter case the subscripts *j* and τ change to τ_*i*_ and τ_*j*_ respectively, since we have to account for the different microbial networks and functions associated to the niche *G*_τ_).

The holobiont error for the specialized interaction case is computed by averaging $\xi_{\tau }^{G}$ over all the niches: $\xi^{L}=\frac{1}{P_{G}}\sum_{\tau =1}^{P_{G}}\xi_{\tau }^{G}$.

## Author Contributions

SH, SS-M, AF, and MA: conceived the experiments, analyzed the data, and wrote and revised the manuscript. SH and MA: designed the experiments. SH: designed the software used in the analysis.

### Conflict of Interest Statement

The authors declare that the research was conducted in the absence of any commercial or financial relationships that could be construed as a potential conflict of interest.
