# Supplementary material for: Modeling the Role of the Microbiome in Evolution
Source: Front Physiol. 2018 Dec 20;9:1836. doi: 10.3389/fphys.2018.01836 (PMC6307544; doi:10.3389/fphys.2018.01836)
Supplement: Supplementary file 6 [file Data_Sheet_1.pdf]

Supplementary Material: Modeling the role of the microbiome in evolution

1 FIGURES

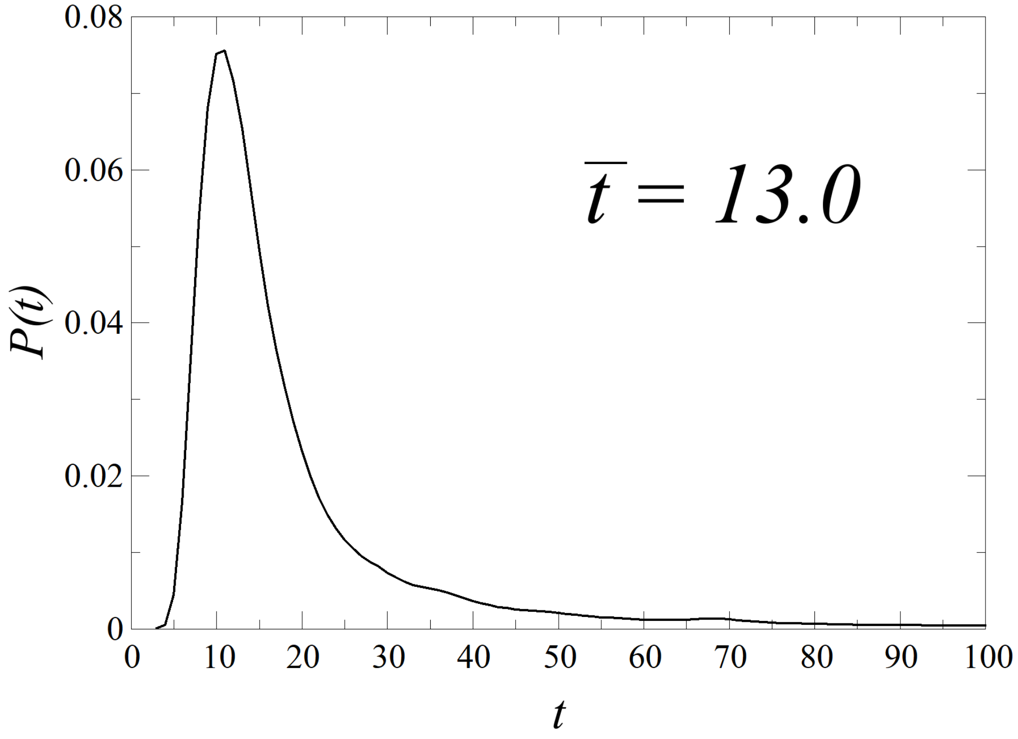

Figure S1. Relaxation time. Distribution $P(t)$ of the relaxation time t to reach the attractor (a periodic pattern of activity) in Kauffman networks with $N = 50$ nodes and connectivity $K = 2$. The average relaxation time is $\bar{t} = 13.0$. The curve was obtained by computing the relaxation time for 100000 networks and probing 100 random initial conditions per network.

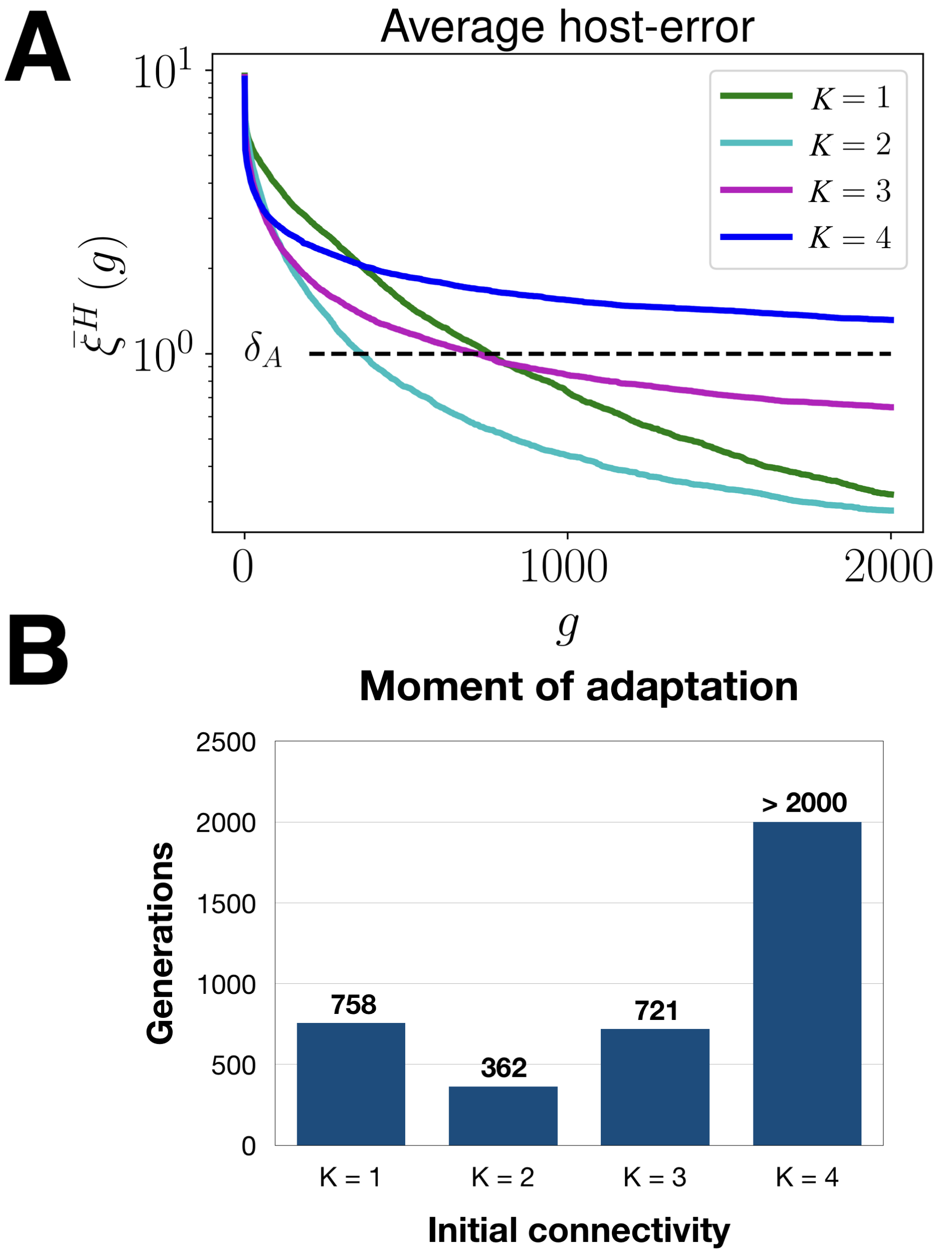

Figure S2. Time required to train networks with different connectivities. (A) Evolution of the host-network error $\xi^H(g)$ throughout generations g for populations of $P = 100$ host networks evolving by themselves, i.e. without interacting with microbial networks. Each network in the population has $N = 50$ nodes and initial connectivity K. The different curves correspond to different values of K. (B) Number of generations elapsed until the host-network error crossed the adaptation threshold δ_A for the different values of the connectivity K. Note that this "adaptation time" is minimum for networks with $K = 2$, whereas for networks with $K = 4$ the adaptation threshold is never crossed within the computing time (2000 generations). These results show that networks with $K = 2$ (which are dynamically critical) are trained faster than networks with connectivity smaller or larger than $K = 2$.

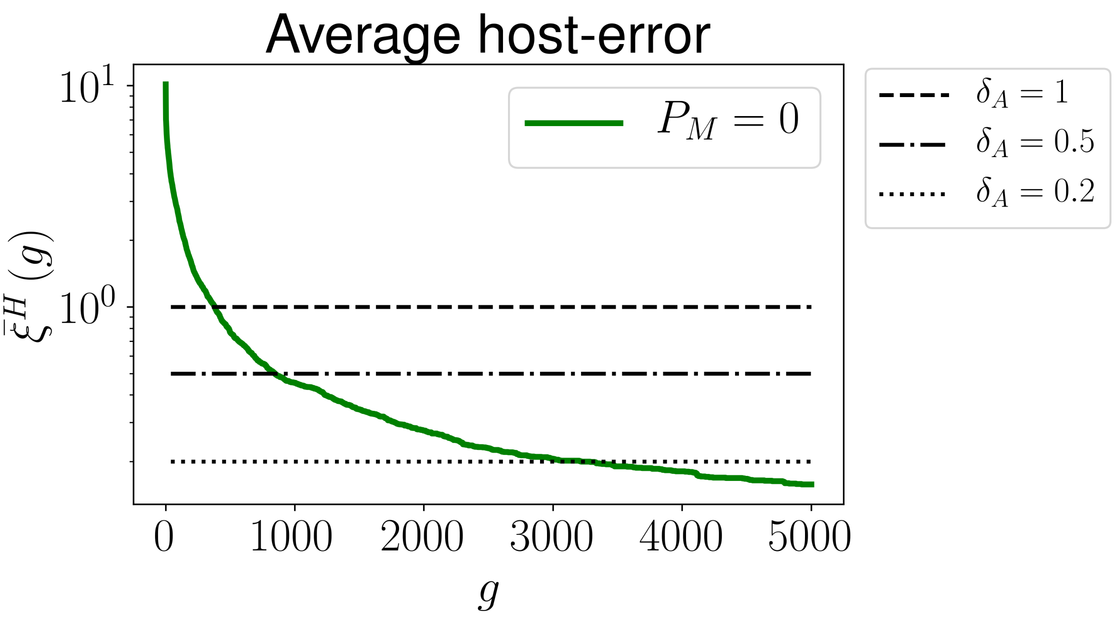

Figure S3. Host network evolving by itself. Graph of the average host-network error ξ^H as a function of generations for the control case (the host network evolving by itself, i.e. without interacting with other networks). This is similar to Fig. 1D of the main text, but over 5000 generations. The broken horizontal lines represent different values of the adaptation threshold δ_A. Note that it takes about 3200 generations for ξ^H to cross the adaptation threshold $\delta_A = 0.2$.

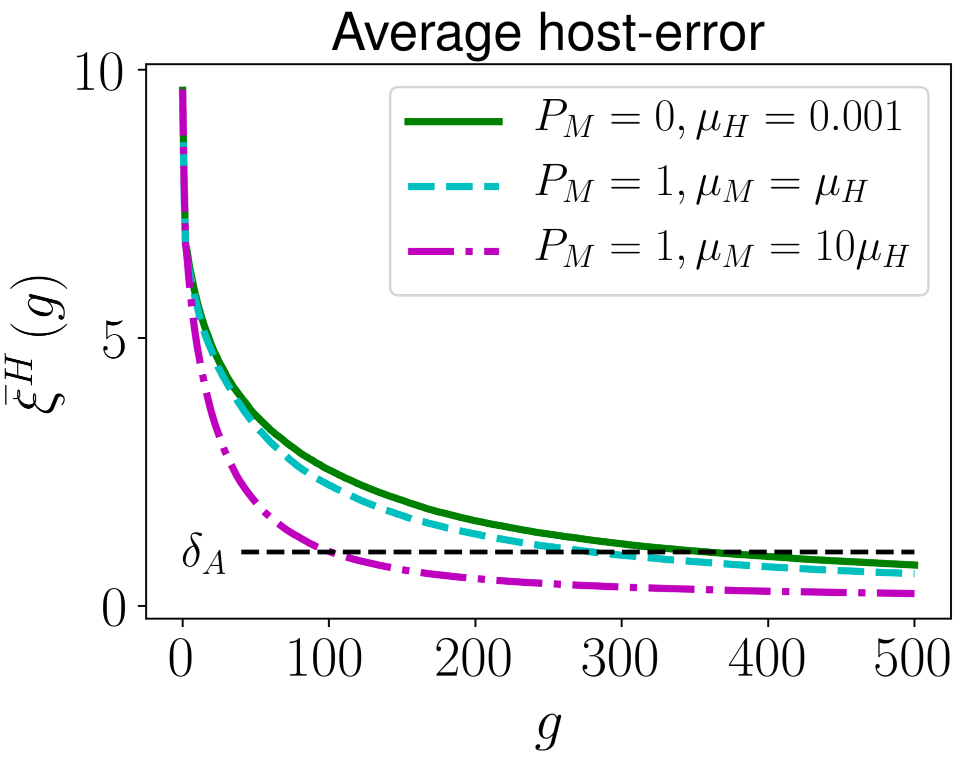

Figure S4. Holobiont evolution with equal mutation rates. Graph of the average host network error ξ^H across generations for the control case (green curve) and a holobiont with $\mu_M = \mu_H = 0.001$ (cyan dashed curve). For reference we also show the case treated in the main text, which is a holobiont with $\mu_M = 10\mu_H = 0.01$ (magenta dotted-dashed curve). Note that when the mutation rates of the host and microbial networks are the same, the interaction with the microbial network has a negligible effect on the adaptation of the host.

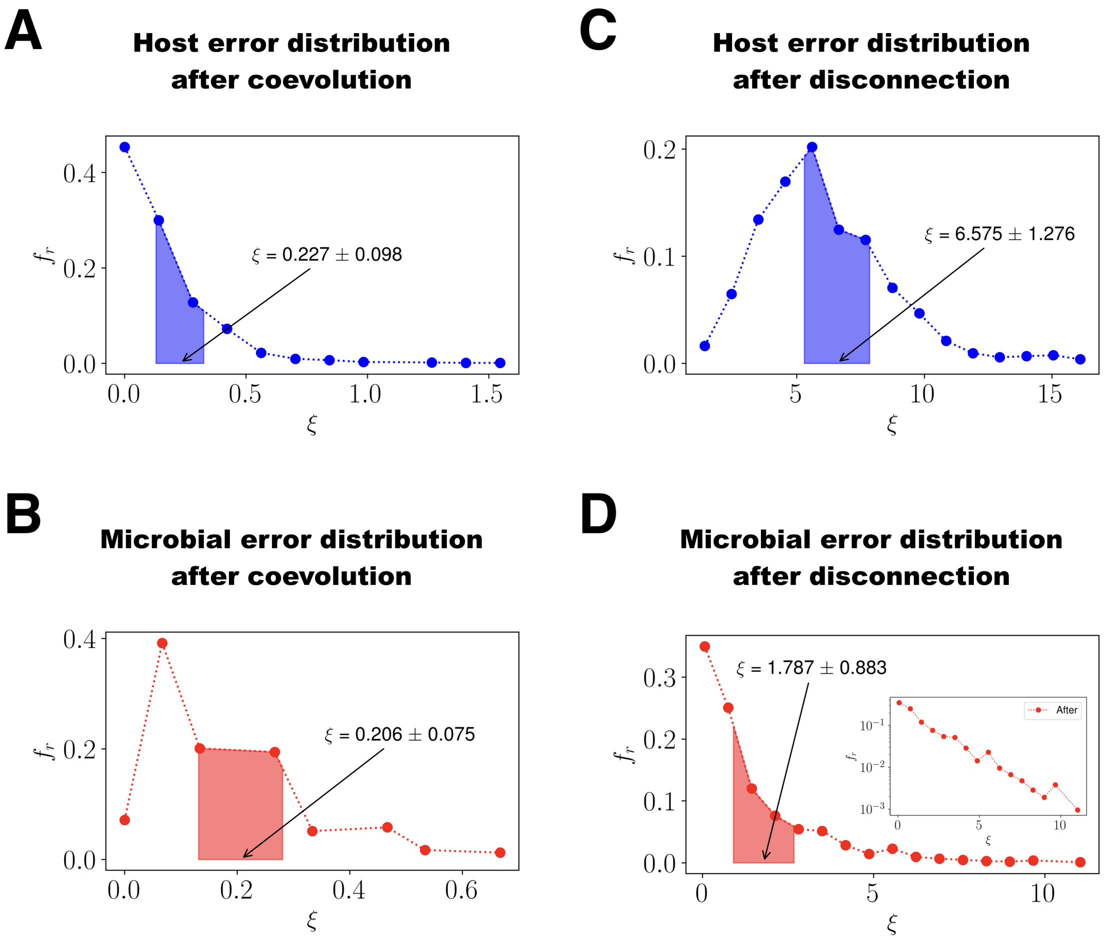

Figure S5. Host-network and microbial-network error distributions. (A) Distribution $f_r\left(\xi^H\right)$ of the host-network error ξ^H in a population of $P = 100$ holobionts that have evolved for 500 generations. The population average is $\bar{\xi}^H = 0.227$ (indicated by an arrow), and very few networks in the population have errors larger than the adaptation threshold $\delta_A = 1$. (B) Distribution $f_r\left(\xi^M\right)$ of the microbial error ξ^M in the same population as in (A). The population average of the microbial error after coevolution for 500 generations is $\bar{\xi}^M = 0.206$, and none of the microbial networks in the population exceeds the adaptation threshold $\delta_A = 1$. (C) Once the holobiont is well adapted (after 500 generations), the host and microbial networks are disconnected. This graph shows the distribution $f_r\left(\xi^H\right)$ of the host-network error after disconnection. Note that the population average increases to $\bar{\xi}^H = 6.575$ and only very few networks remain below the adaptation threshold $\delta_A = 1$. (D) Distribution $f_r\left(\xi^M\right)$ of the microbial error ξ^M after disconnecting the host and microbial networks in the same population as in (C). After disconnection, the average microbial error in the population increases to $\bar{\xi}^M = 1.787$ which is above the adaptation threshold $\delta_A = 1$. The inset is a semi-logarithmic plot of the distribution $f_r\left(\xi^M\right)$, showing that after disconnection most of the microbial networks in the population increase their errors way beyond the adaptation threshold.

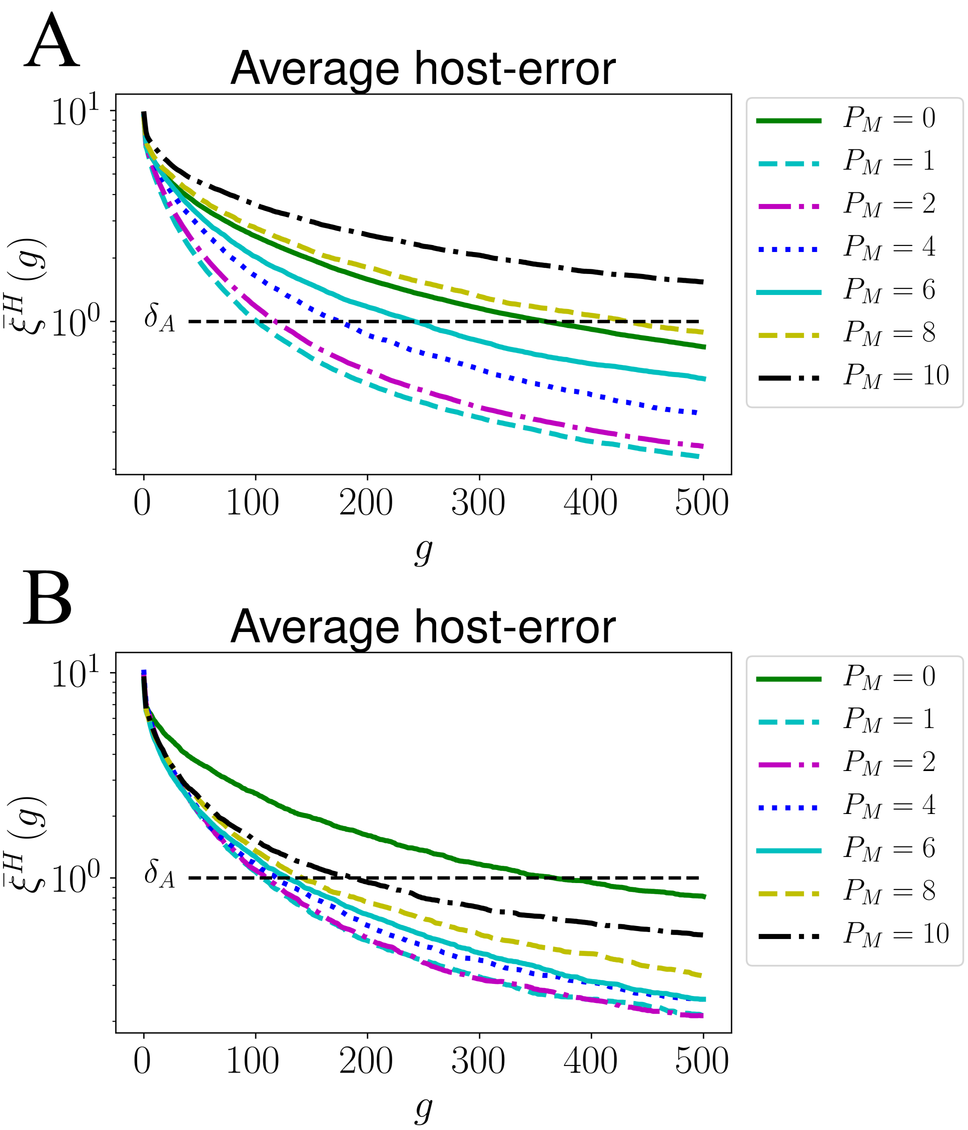

Figure S6. Evolutionary conflict in the holobiont. (A) Graphs of the evolution of the average host-network error $\bar{\xi}^H$ across generations for holobionts with many microbial networks. The host network has to perform only one task, and during its adaptation it interacts with P_M microbial networks. The different curves correspond to different values of P_M, ranging from $P_M = 0$ (the host network evolving by itself) to $P_M = 10$. Note that, except for the case $P_M = 1$, the more microbial networks in the holobiont, the worse the adaptation of the host network. When the host network has to perform only one task, and many microbial networks participate in its adaptation, evolutionary conflict appears within the holobiont, as the microbial networks obstruct each other in helping the host network to adapt to its task. For $P_M = 10$ the host's adaptation is even worse than letting it evolve by itself. (B) Same type of data as before but using the alternative definition for the holobiont error ξ^L given in Eq. (5) of the main text. In this case, the host network and the entire microbiota have the same contribution to the holobiont error regardless of the number of microbial networks in the microbiota. Qualitatively, the same evolutionary conflict emerges using either the definition of ξ^L given in Eq. (4) or the one given in Eq. (5) of the main text. This indicates that our results are not very sensitive to the particular details of the definition of the error function through which the adaptation of the holobiont is determined.

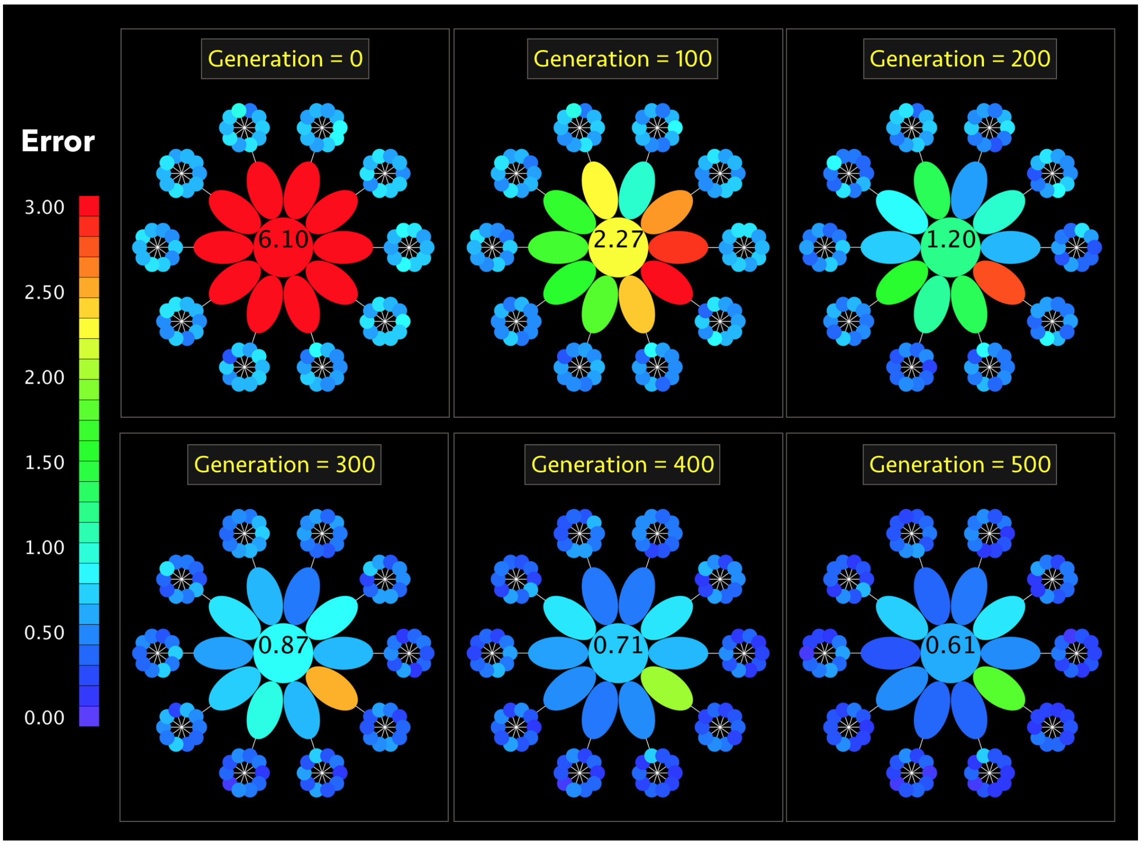

Figure S7. Holobiont adaptation with 100 specialized microbial networks. This figure shows snapshots of the evolution of a holobiont in which the host network has to adapt to $T = 10$ tasks and is being assisted by $P_M = 100$ microbial networks distributed across 10 niches. The host network is represented by the big circle at the center; the microbial networks are represented by small circles in the periphery; the tasks are represented by ellipses. The color code shows the error. All errors larger than 3 are colored in red. After 300 generations the average host-network error is already below the adaptation threshold: $\bar{\xi}^H = 0.87$. This shows that, as long as the microbial networks are structured into specialized niches, they do not generate evolutionary conflict within the holobiont. However, one niche will require more than 500 generations to fully adapt to its task (the green one in the last snapshot). It is clear that the different niches adapt to their respective tasks at different rates.

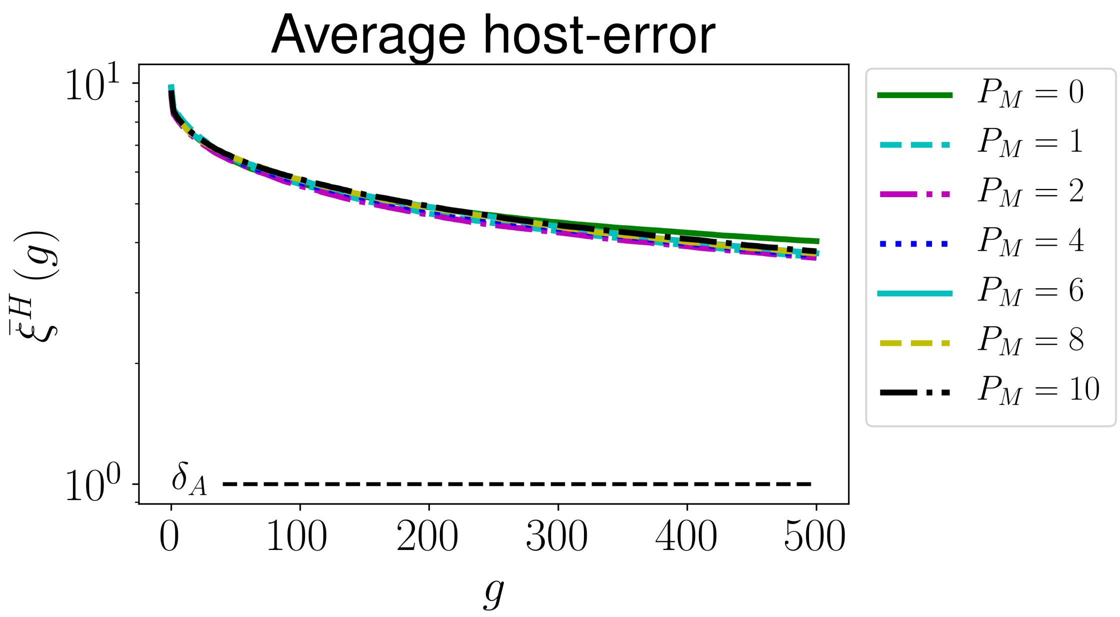

Figure S8. The holobiont as one big evolving network. When the mutation rates of the host and microbial networks are the same, i.e. $\mu_M = \mu_H = 0.001$, and all the microbial networks equally participate in the adaptation of the host network to all of its tasks (non-specialized case), there is neither dynamical nor structural organization within the holobiont. The holobiont can thus be considered as just one big homogeneous network trying to evolve in order to perform many tasks. This graph shows the average host-network error $\bar{\xi}^H$ across generations for this homogeneous case. Each curve corresponds to a different number P_M of microbial networks assisting the host to adapt to $T = 10$ different tasks. The number of microbial networks ranges from $P_M = 0$ to $P_M = 10$. It is clear that interacting with a non-specialized microbiota which, in addition, generates mutants at the same rate as the host, has no effect whatsoever on the adaptation of the holobiont, regardless of the number of microbial networks. (Compare with Fig. S4.)

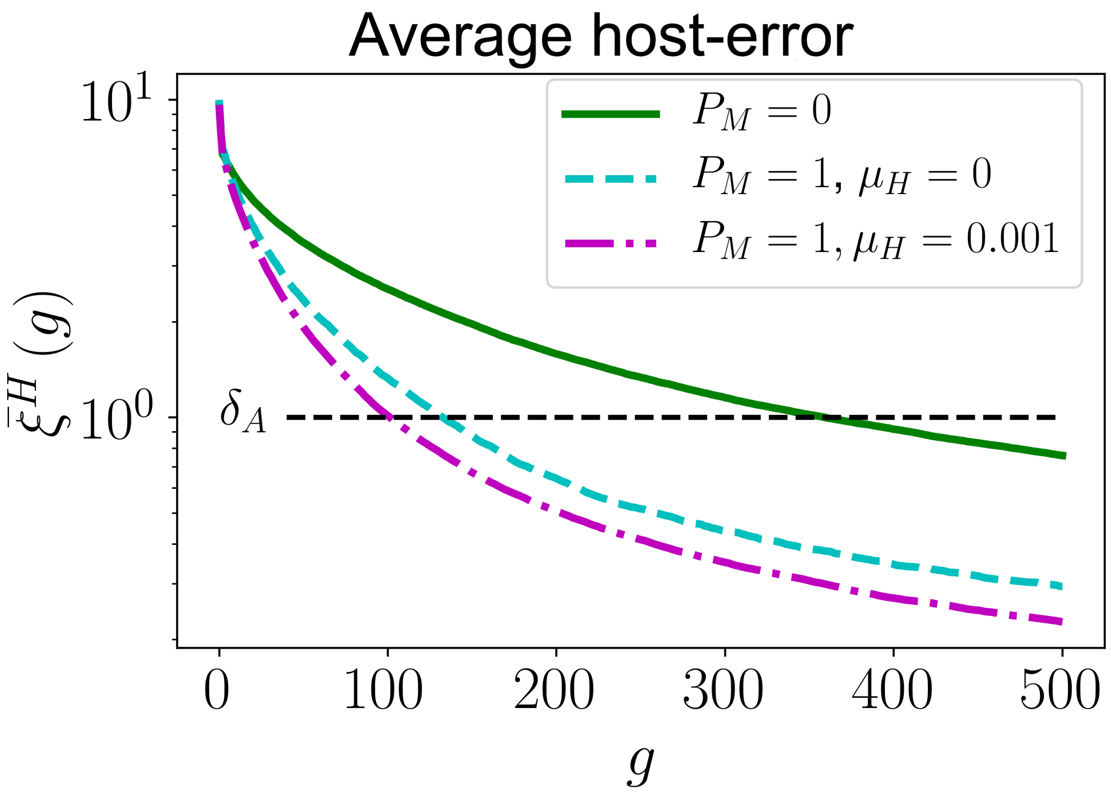

Figure S9. Host's adaptation within its lifespan. Average host-network error $\bar{\xi}^H$ across generations for a holobiont with one host network and one microbial network. The green curve corresponds to the control case. The dashed curve (cyan) corresponds to the case in which the host network does not mutate ($\mu_H = 0$), but only the microbial network. Throughout the evolutionary processes, regulatory interactions from the microbial network to the host network are allowed to emerge. In this case, the adaptation of the host network to its task is entirely due to regulations coming from the microbial network. In biological terms, this could be interpreted as the host moving to a new environment and acquiring a new microbiota, which will help the host to adapt to the new environment within the host's lifespan. The dotted-dashed curve (magenta) corresponds to the usual case discussed in the main text in which both the host and the microbial networks mutate with the rates $\mu_H = 0.001$ and $\mu_M = 0.01$, respectively. Note that even in the extreme case $\mu_H = 0$, interaction with the microbial network greatly improves and accelerates the adaptation of the host network with respect to the control case.

2 MOVIES

Movie S1

Evolution of the host network. Typical example of the evolution of one host network evolving by itself, i.e., without interacting with any other network. The target function $F(t)$ is the white curve, whereas the network's output signal $R(t)$ is the green curve. Note that at the beginning of the simulation $F(t)$ and $R(t)$ are quite different. However, as the evolution proceeds, $R(t)$ approaches the task $F(t)$. The top of the left panel shows the number of generations, the mutation rate, the number of accumulated mutations in the network and the value of the error ξ^H. At the bottom of the left panel we show the graph of the evolution of ξ^H across generations. In this example the error ξ^H crosses the adaptation threshold $\delta_A = 1$ at generation $g = 375$. The white dashed line in the graph indicates the adaptation threshold. The network has $N = 50$ nodes and $N_s = 12$ signal nodes.

Movie S2

Holobiont evolution: one microbial network. Typical example of the evolution of a holobiont consisting of one host network (green) and one microbial network (red). For both networks the white curves represent the corresponding tasks, whereas the green and red curves are the output signals of the host and microbial networks, respectively. Note that the microbial network is well adapted to its task from the very beginning. Throughout the evolution of the holobiont, regulatory interactions emerge between the host and microbial networks (broken lines between the networks). The bottom of the left panel shows the graph of the host (green) and microbial (red) errors across generations. The white dashed line in the graph indicates the adaptation threshold $\delta_A = 1$. Each network has $N = 50$ nodes and $N_s = 12$ signal nodes.

Movie S3

Holobiont evolution: two microbial networks. Typical example of the evolution of a holobiont consisting of one host network (green) and two microbial networks (red and blue). For all networks the white curves represent the corresponding tasks, whereas the green, red and blue curves are the output signals of the host and microbial networks, respectively. At the start of the simulation the microbial networks are already well adapted to their tasks. Throughout the evolution of the holobiont, regulatory interactions emerge between all the networks (broken lines). The bottom of the left panel shows the graph of the host (green) and microbial (red and blue) errors across generations. The white dashed line in the graph indicates the adaptation threshold $\delta_A = 1$. Each network has $N = 50$ nodes and $N_s = 12$ signal nodes.

Movie S4

Holobiont evolution: specialized interaction. Typical example of the evolution of a holobiont whose host network has to perform $T = 10$ different tasks (represented as ellipses). There are $P_M = 25$ microbial networks (small circles) distributed across 10 niches, which contain either two or three microbial networks. The host network is the big circle at the center. The color code indicates the value of the error, with all errors larger than 3 colored in red. Note that initially all the microbial networks are well adapted. As the evolution of the holobiont proceeds, the host network adapts to all of its tasks, although at different rates

for each task. The number on the host network represents its average error over all the tasks. The holobiont crosses the adaptation threshold $\delta_A = 1$ at generation $g = 270$.

Movie S5

Holobiont evolution: microbial network with no task. Typical example of the evolution of a holobiont with one host network (green) and one microbial network (red). In this case, the microbial network is free to evolve without having to perform any task. Therefore, there is no selective pressure on the microbial network other than to contribute to the adaptation of the host network. The white curve under the host network is the task it has to perform, whereas the green curve is its output signal. The red curve is the microbial network's output signal, which in this case is free of any restriction. Note that initially the output signal of the microbial network abruptly changes from one generation to the next. However, as the evolution proceeds, the output signal of the microbial network stabilizes and does not change any more. This is because the microbial network acquires regulatory interactions from and to the host network that stabilizes the dynamics of the entire holobiont in order for the host network to achieve its task.
